# Acetylation at Lysine 86 of *Escherichia coli* HUβ Modulates the DNA-Binding Capability of the Protein

**DOI:** 10.3389/fmicb.2021.809030

**Published:** 2022-02-04

**Authors:** Victoria L. Barlow, Yu-Hsuan Tsai

**Affiliations:** ^1^School of Chemistry, Cardiff University, Cardiff, United Kingdom; ^2^Institute of Molecular Physiology, Shenzhen Bay Laboratory, Shenzhen, China

**Keywords:** lysine acetylation, DNA-binding protein, HU, *Escherichia coli*, genetic code expansion

## Abstract

DNA-binding protein HU is highly conserved in bacteria and has been implicated in a range of cellular processes and phenotypes. Like eukaryotic histones, HU is subjected to post-translational modifications. Specifically, acetylation of several lysine residues have been reported in both homologs of *Escherichia coli* HU. Here, we investigated the effect of acetylation at Lys67 and Lys86, located in the DNA binding-loop and interface of *E. coli* HUβ, respectively. Using the technique of genetic code expansion, homogeneous HUβ(K67ac) and HUβ(K86ac) protein units were obtained. Acetylation at Lys86 seemed to have negligible effects on protein secondary structure and thermal stability. Nevertheless, we found that this site-specific acetylation can regulate DNA binding by the HU homodimer but not the heterodimer. Intriguingly, while Lys86 acetylation reduced the interaction of the HU homodimer with short double-stranded DNA containing a 2-nucleotide gap or nick, it enhanced the interaction with longer DNA fragments and had minimal effect on a short, fully complementary DNA fragment. These results demonstrate the complexity of post-translational modifications in functional regulation, as well as indicating the role of lysine acetylation in tuning bacterial gene transcription and epigenetic regulation.

## Introduction

Histone-like protein HU is a prevalent DNA-binding protein ubiquitous among bacterial species (Grove, 2011). This basic protein is highly conserved, consisting of an α-helical “body” and two β-sheets that are extended to β-ribbon “arms” interacting directly with DNA (Swinger et al., 2003). Through its interaction with DNA, HU is associated with bacterial phenotypes, including survival and virulence. For example, HU knockout in Gram-positive bacteria is lethal due to disruption in genome integrity (Micka and Marahiel, 1992; Bartels et al., 2001; Liu et al., 2008; Nguyen et al., 2009). HU can also modulate the pathogenicity of different bacteria through transcriptional regulation (Alberti-Segui et al., 2010; Koli et al., 2011; Mangan et al., 2011; Wang et al., 2014; Phan et al., 2015).

The majority of bacterial species produce one HU homolog, which forms homodimers (Grove, 2011). However, like other enterobacteria, *Escherichia coli* has two HU homologs: HUα and HUβ, encoded by *hupA* and *hupB* genes, respectively. Consequently, three distinct dimeric forms of HU exist. HUα and HUβ can each form homodimers (HUα_2_ and HUβ_2_) alongside a heterodimer (HUαβ). The proportion of each dimer changes throughout the cell cycle, and HUαβ predominates in the late exponential and stationary phase (Claret and Rouviere-Yaniv, 1997).

In *E. coli*, HU is one of the most abundant proteins (ca. 30,000 dimers per cell) and randomly distributes across the genome (Prieto et al., 2011; Wang et al., 2011). While HU does not display any sequence specificity and is capable of binding both single- and double-stranded DNA, it has higher affinity to double-stranded DNA with structural deformities such as gaps, nicks, etc. (Kamashev and Rouviere-Yaniv, 2000). In addition, the heterodimer displays higher affinity for DNA than either homodimer (Castaing et al., 1995; Pinson et al., 1999). Further, when bound to DNA, HU can induce flexible bends of 10–180° (Rice et al., 1996; Swinger et al., 2003; Swinger and Rice, 2004; Becker et al., 2005).

Like eukaryotic histones, *E. coli* HU is also subjected to post-translational modifications. Specifically, acetylation at several lysine residues has been reported in both homologs (Zhang et al., 2013; Castaño-Cerezo et al., 2014). However, the functional implication of these site-specific modifications remains largely elusive. We previously characterized the effect of site-specific acetylation on HU of nosocomial pathogen *Acinetobacter baumannii*, which has only one homolog encoded by *hupB* (Liao et al., 2017). We reported acetylation of *A. baumannii* HU at Lys13, which is also conserved in many bacterial HU homologs, including *E. coli* HUα. Functionally, Lys13 acetylation altered both the thermal stability and DNA binding kinetics of *A. baumannii* HU, while no changes were observed in either the secondary structure, oligomeric state, or affinity to DNA. Since we are interested to know whether lysine acetylation may act as an epigenetic regulator in bacteria, we decided to investigate if modification of a lysine residue in the DNA-binding site can modulate the affinity of HU to DNA.

To this end, we chose *E. coli* HUβ as the model protein for investigating the effect of acetylation at Lys67 and Lys86, located in the DNA binding-loop or interface, respectively. Using the technique of genetic code expansion (Chen et al., 2018; Nödling et al., 2019; Chen and Tsai, 2021), homogeneous HUβ with acetylation at Lys67 (K67ac) or Lys86 (K86ac) were produced recombinantly in *E. coli*. We found that acetylation at Lys86 of HUβ can regulate the affinity of the HU homodimer, but not the heterodimer, to DNA. Intriguingly, while HUβ(K86ac)_2_ has a lower affinity than that of the unmodified HUβ_2_ to short (i.e., 30 bp) double-stranded DNA fragments containing a 2-nucleotide gap or nick, HUβ(K86ac)_2_ binds with higher affinity than HUβ_2_ to longer (>1000 bp) DNA fragments. The results demonstrate the complexity of bacterial post-translational modification in functional regulation, as well as indicating that lysine acetylation may fine tune gene transcription in bacteria and be involved in epigenetic regulation.

## Materials and Methods

### Homology Modeling

Amino acid sequence of *E. coli* HUβ (Swiss-Prot/TrEMBL accession number P0ACF4) was used to build the DNA-bound homology model by SWISS-MODEL (Biasini et al., 2014; Bienert et al., 2016) using PDB 1P51 (*Anabaena* HU) as the template. The model was visualized using UCSF Chimera (Pettersen et al., 2004).

### Plasmid Construction

All plasmids were propagated in *E. coli* Stbl3, and their sequences were confirmed by Sanger sequencing. Plasmid pCDF AcKST expresses an orthogonal aminoacyl-tRNA synthetase/tRNA_*CUA*_ pair (Neumann et al., 2009) for acetyl lysine incorporation and has been described previously (Liao et al., 2017).

For construction of plasmid pBAD hupA-His6, chromosomal DNA of *E. coli* BL21(DE3) was isolated and used as the PCR template to amplify *hupA* with primers CAGCTGCAGATCTCAGTGGTGGTGGTGGTGGTGCTTAAC TGCGTCTTTCAGTGCC and GGCTAACAGGAGGAA TTAACATGAACAAGACTCAACTGATTGATGTAATTGC. The *hupA* gene was then cloned into a pBAD vector amplified with primers ACCACTGAGATCTGCAGCTG and CATGTTAATTCCTCCTGTTAGC by Gibson assembly.

For construction of plasmid pBAD hupB-His6, chromosomal DNA of *E. coli* BL21(DE3) was isolated and used as the PCR template to amplify *hupB* with primers GGCTAACAGGAGGAATTAACATGAATAAATCTCAATTGAT CGACAAGATTGCTGC and TATGGTACCAGCTGCA GATCGTGGTGGTGGTGGTGGTGCTCGAGGTTTACCGCGT CTTTCAGTGCTTTACC. The *hupB* gene with a C-terminal His-tag was then cloned into pBAD vector between the *Nco*I and *Xho*I sites by Gibson assembly to provide the title plasmid.

Construction of plasmid pBAD hupB(K67TAG)-His6 was achieved by amplification of pBAD hupB-His6 with primers GCAGCAGCGATGGTGATCTCCTAACCGGTCTGCGGGTTG C and GAGATCACCATCGCTGCTGCTAAAGTAC. The resulting PCR product was circularized by homologous recombination in *E. coli* Stbl3. Plasmid pBAD hupB(K86TAG)-His6 was constructed in a similar fashion with primers TGCTCGAGGTTTACCGCGTCCTACAGTGCTTTACCTGCAC GGAAGC and GACGCGGTAAACCTCGAGCAC.

### Recombinant Protein Production and Purification

pBAD hupA-His6 and pBAD hupB-His6 were chemically transformed into *E. coli* BL21-AI. For acetyl lysine incorporation, pBAD hupB(K67TAG)-His6 or pBAD hupB(K86TAG)-His6 were co-transformed with pCDF AcKST. The transformation mixture was used to inoculate a starter culture in terrific broth (TB) medium supplemented with 100 μg/mL ampicillin, the selection marker of pBAD, and incubated at 37°C and 180 rpm for 16–18 h. For acetyl lysine incorporation, the medium was also supplemented with 100 μg/mL spectinomycin, the selection marker of pCDF. The starter culture was diluted to OD_600_ 0.05 in 1 L TB supplemented with the appropriate antibiotics and incubated at 37°C and 180 rpm. For acetyl lysine incorporation experiments, at OD_600_ 0.5 the culture was supplemented with 5 mM acetyl lysine and 20 mM nicotinamide, a deacetylase inhibitor. At OD_600_ 0.9 or 30 min after amino acid supplementation, protein expression was induced with 0.5 mM isopropyl β-D-1-thiogalactopyranoside and 0.2% L-arabinose. Cultures were incubated at 20°C and 180 rpm for 16–18 h.

Cultures were centrifuged at 4500 × *g* for 20 min at 4°C. The supernatant was discarded, and pellets were resuspended in 10 mL chilled lysis buffer (0.05 M Tris–HCl pH 8.0, 0.15 M NaCl, 0.01 M imidazole, 1 g/L lysozyme, 100 μM PMSF) per 1 g cell pellet. Cells were sonicated (Sonics, Vibra-Cell™) on ice in bursts of 5-s ON and 15-s OFF using a 13 mm probe (Sonics) at 39% amplification until lysed. The lysate was then centrifuged at 30,000 × *g* and 4°C for 20 min. The supernatant was collected and passed through a 0.44 μm syringe filter, then combined with Ni-NTA resin equilibrated in lysis buffer and incubated at 4°C with gentle agitation for 1–2 h. The lysate/resin was poured into a gravity column, and the flow through was collected. The resin was washed with chilled wash buffer (0.05 M Tris–HCl pH 8.0, 0.15 M NaCl, 0.02 M imidazole) until no protein could be detected in the flow through by a NanoDrop One (ThermoFisher, #ND-ONE-W) measuring protein A_280_ (1 Abs = 1 mg/mL). The protein was then eluted from the column in 1 column volume fractions using chilled elution buffer (0.05 M Tris–HCl pH 8.0, 0.15 M NaCl, 0.25 M imidazole) and analyzed by 20% SDS-PAGE.

Fractions containing protein at the estimated molecular weight were exchanged into potassium phosphate buffer (10 mM KPO, pH 7.0, 5% glycerol) using a HiPrep 26/10 desalting column (Cytiva, #17-5087-01) per manufacturer’s instructions. The sample was then loaded onto a RESOURCE S column (Cytiva, #17-1180-01) and eluted in phosphate buffer with a 0 to 0.5 M NaCl gradient. In both instances, protein elution was monitored at 214 nm. Fractions containing a peak at 214 nm were analyzed by SDS-PAGE before being pooled and concentrated using a Vivaspin 20, 3000 MWCO PES (Sartorius, #VS2091). Protein was dialyzed using 3500 MWCO SnakeSkin dialysis tubing in at least 1000 times the protein sample volume of phosphate buffer at 4°C for 16–18 h. Protein concentration was determined by BCA assay. For storage at –80°C, final glycerol concentration was adjusted to 10%.

The molecular weight of all protein samples was analyzed using mass spectrometry by Cardiff University School of Chemistry Analytical Services. Mass spectra were acquired on an Waters Acquity H-Class UPLC system coupled to a Waters Synapt G2-Si quadrupole time of flight mass spectrometer with a Waters Acquity UPLC Protein C4 BEH column 300 Å, 1.7 μm (2.1 × 100 mm).

To produce the heterodimers, purified HUα_2_ was combined with HUβ_2_, HUβ(K67ac)_2_ or HUβ(K86ac)_2_ in a 1:1 molar ratio and incubated on ice for 5 min (Ramstein et al., 2003). The resulting heterodimers were confirmed *via* analytical size exclusion chromatography.

### Analytical Size Exclusion Chromatography

Analytical size exclusion chromatography was performed with a high-performance liquid chromatography system (Agilent). A Bio SEC-3 column (Agilent) was equilibrated in PBS (10 mM NaPO pH 7.4, 154 mM NaCl). For generating the standard curve of molecular weights, Gel Filtration Standard (Bio-Rad, #1511901) was diluted 1:10 in potassium phosphate buffer and 1 μL of the diluted standard was injected into the column using an autosampler. PBS was passed through the column at a rate of 0.35 mL/min and the elution profile of the standards was analyzed at 214 nm. For analyses of HU protein, protein samples were prepared at 0.6 mg/mL and 20 μL of sample was injected into the column using an autosampler. PBS was passed through the column at a rate of 0.35 mL/min and the elution profile of the proteins were analyzed at 214 nm.

For the standard curve, the log molecular weights of the protein standards were plotted against their retention time and a line of best fit generated. The equation of the line of best fit was then used to calculate an estimated molecular weight of the HU protein samples.

### Electrophoretic Mobility Shift Assay

For electrophoretic mobility shift assays with 30 bp fragments, custom oligonucleotides (Merck) were purified by high pressure liquid chromatography and provided in TE buffer at 100 μM concentration. Mixing individual oligonucleotides yielded three double-stranded DNA fragments: a 30-bp duplex with a 2 nucleotide gap, a 30-bp duplex with a nicked phosphate backbone, and a 30-bp complete duplex (Castaing et al., 1995). Annealing of the 30-bp fragments was ensured by heating combined oligonucleotides to 98°C for 5 min, then cooling to 4°C at a rate of 1°C/min.

Oligonucleotides used for constructing the 30-bp duplex with a 2-nucleotide gap: CCAACTTCCCTA ACCCAGCTGCGATCCGTA, TACGGATCGCAGC and GGTTAGGGAAGTTGG; for 30-bp duplex with a nicked phosphate backbone: CCAACTTCCCTAACC CAGCTGCGATCCGTA, TACGGATCGCAGC and TGGGTTAGGGAAGTTGG; for the 30-bp complete duplex: CCAACTTCCCTAACCCAGCTGCGATCCGTA and TACGGATCGCAGCTGGGTTAGGGAAGTTGG.

Each reaction contained 1 μM of a 30 bp DNA fragment, varied concentrations of HU protein and binding buffer (30 mM Tris pH 7.5, 100 mM NaCl, 0.02% v/v Tween20, 0.5 mg/ml BSA) in a 10 μL reaction volume and was incubated at 18°C for 10 min. After incubation, 2.5 μL of GelPilot DNA Loading Dye (Qiagen, #239901) was added to the samples and the entire sample electrophoresed on a 10% non-denaturing acrylamide gel in TBE buffer (100 mM Tris-borate pH 8.3, 2 mM EDTA) polymerized with a final concentration of 0.2% ammonium persulfate and 0.1% tetramethylethylenediamine. Gels were electrophoresed on ice in TBE for 80 min before DNA was visualized using PAGE GelRed (Biotium, #41008) per manufacturer’s instructions. Gels were imaged on a Bio-Rad ChemiDoc MP system.

For assays with longer DNA fragments, linearized plasmid, pCX eGFP (Perry et al., 1999; Liao et al., 2017) or pUC18 (Thermo Scientific, #SD0051), was produced through restriction enzyme digestion with *Hin*dIII (Thermo Scientific, #FD0504). A 1292-bp PCR fragment was obtained by PCR of the gene encoding maltose binding protein with primers TTTTGTTTAACTTTAAGAAGGAGATATACATATGAAAATA AAAACAGGTGCACGCATCC and CCTGAAAATA AAGATTCTCGCTAGCCCTTCCCTCGATCCCGAGGTTG. The full DNA sequences of the 5500, 2700, and 1292 bp DNA fragments are provided in the Supplementary Material.

Each reaction contained 250 ng of DNA, varied concentrations of HU protein and binding buffer in a 15 μL reaction volume and was incubated at 37°C for 1 h (Ghosh et al., 2016; Liao et al., 2017). After incubation, 3.75 μL of GelPilot DNA Loading Dye was added to the samples and the entire sample electrophoresed on a 0.5% agarose gel containing SYBR Safe (ThermoFisher) in 1× TAE (40 mM Tris-acetate pH 8.0, 1 mM EDTA) buffer on ice. Gels were imaged on a Bio-Rad ChemiDoc MP system.

### DNA Supercoiling Assay

DNA supercoiling assays were performed as described by Guo and Adhya (2007). Briefly, plasmid pCX eGFP was relaxed with *E. coli* topoisomerase I (New England Biolabs) according to manufacturer’s instructions. Then, 100 ng of relaxed DNA was prepared in 1× topoisomerase buffer (TaKaRa Bio), 0.01% BSA and incubated with 0.5 μg of HUβ_2_ or HUβ(K86ac)_2_ in a 10 μL reaction. The reaction was incubated at 37°C for 30 min. Then, 7 units of calf thymus topoisomerase I (TaKaRa Bio) was added to each reaction and the reactions incubated at 37°C for 2 h. 10 μg of proteinase K (Invitrogen) was added to each reaction, which were incubated at 37°C for 30 min. Then, 5× DNA loading dye (Qiagen, #239901) was added to each reaction and the samples were analyzed on 0.8% agarose in 1× TBE buffer and electrophoresed at 150 V for 90 min. Post-run, the gel was stained with SybrSafe (Invitrogen) according to manufacturer’s instructions and imaged on a Bio-Rad ChemiDoc MP system.

### Circular Dichroism Spectroscopy

These experiments were performed on an Applied PhotoPhysics Chirascan spectrometer using 5 μM protein in deoxygenated potassium phosphate buffer (10 mM KPO, pH 7.0, 5% glycerol). Spectra were measured in triplicate between 200 and 300 nm in 1 mm quartz cuvettes under N_2_ with a 50 nm/min scan speed, 0.5 nm data pitch, 1 nm bandwidth, and 0.5 s response time. To measure the thermal melting point, spectra were collected every 2°C as temperature increased from 4 to 84°C. The rate of temperature increase was 0.5°C/min with 300 s equilibration time at each temperature.

### Experimental Design and Statistical Rationale

For electrophoretic mobility shift assay, a representative gel from triplicates was shown for each condition. Images of the other two independent repeats are provided in the Supplementary Material. Free DNA remaining in each condition was quantified using ImageLab (Bio-Rad) software with the band intensity of the control lane (DNA only) as the reference (100%). Mean and standard deviation of free DNA remaining calculated from the three experiments are presented. Tables presenting the raw values are provided in the Supplementary Material. Statistical significance between groups was analyzed using an independent two sample *t*-test with an alpha level of 0.05.

## Results

### Identification of Lysine Residues of Which Acetylation May Disrupt Interaction With DNA

**FIGURE 1:**
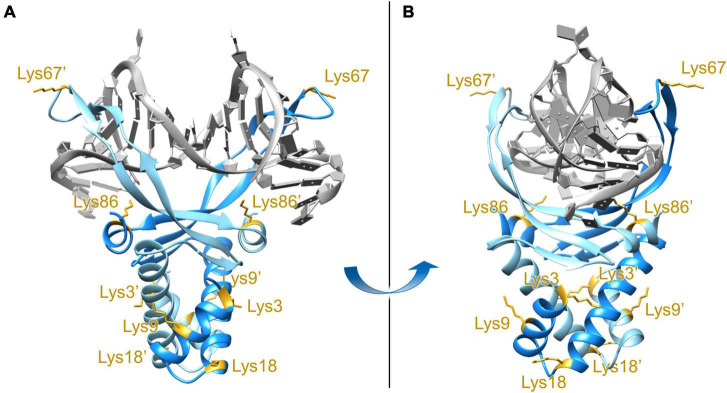
Front view **(A)** and side view **(B)** of the DNA-bound *E. coli* HUβ_2_ homology model. The model was generated using SWISS-MODEL with the amino acid sequence of *E. coli* HUβ_2_ (Swiss-Prot/TrEMBL accession number P0ACF4) and PDB 1P51 (*Anabaena* HU) as the template. Lysine residues at position 3, 9, 18, 67, and 86 have been reported to be acetylated *in vivo* (Castaño-Cerezo et al., 2014) and are annotated in the two subunits. Lys67 and Lys86, located in the DNA-binding β-ribbon arms and interface, respectively, were chosen for further investigation.

While the high-resolution crystal structure of *E. coli* HUβ_2_ is available (pdb: 4P3V), attempts to obtain the structures of HUβ_2_ or HUα_2_ in a DNA-bound state have not yet been successful. Nevertheless, a DNA-bound structure of *Anabaena* HU (pdb: 1P51; Swinger et al., 2003), which shares 42% sequence identity and 71% sequence similarity to *E. coli* HUβ, is available and was employed to generate a homology model using SWISS-MODEL (Biasini et al., 2014; Bienert et al., 2016). Based on the homology model, the β-ribbon arms (amino acid residues 56–73) and the C-terminal α-helix (amino acid residues 82–90) are involved in DNA binding (Figure 1), and this assertion is supported by previous biochemical studies of *E. coli* HU (Bhowmick et al., 2014; Hammel et al., 2016; Agapova et al., 2020; Thakur et al., 2021). A report of *E. coli* acetylome revealed acetylation at Lys3/9/18/67/86 of HUβ (Castaño-Cerezo et al., 2014). As Lys67 (K67ac) and Lys86 (K86ac) are located on the β-ribbon arms and C-terminal α-helix, respectively, we decided to investigate if acetylation at either of these residues changes the properties and/or function of *E. coli* HUβ.

### Production of *E. coli* HUα, HUβ, HUβ(K67ac) and HUβ(K86ac)

To investigate the effects of *E. coli* HUβ acetylation, four variants of HU monomers, HUα, HUβ, HUβ(K67ac) and HUβ(K86ac), were expressed recombinantly in *E. coli* and purified. Using these monomers, we were able to obtain their homodimers, as well as heterodimers of HUαβ, HUαβ(K67ac) and HUαβ(K86ac) since mixing of purified *E. coli* HUα_2_ and HUβ_2_ in a 1:1 ratio *in vitro* leads to spontaneous rearrangement to afford the heterodimer (Ramstein et al., 2003).

**FIGURE 2:**
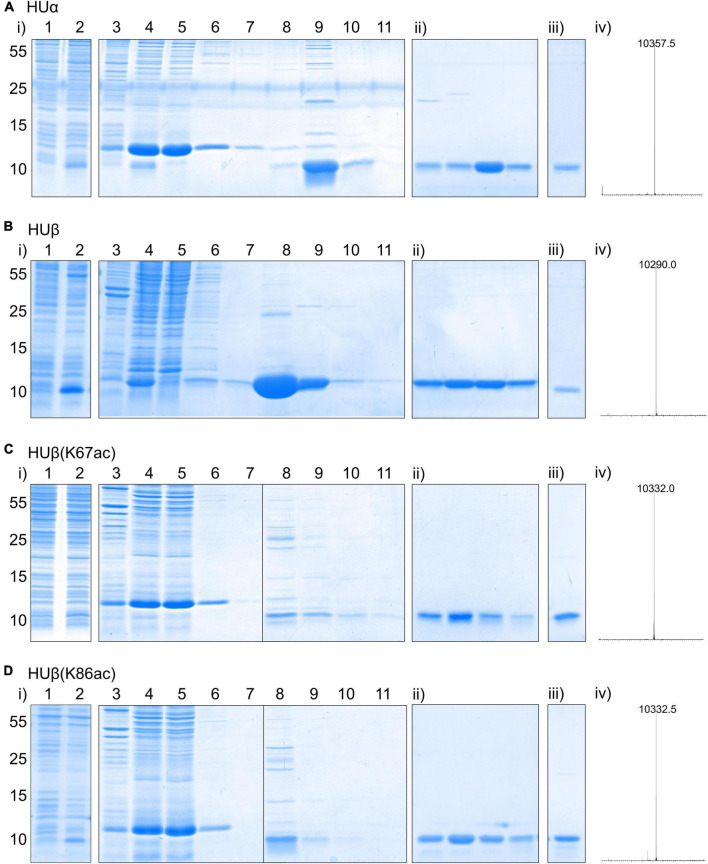
Expression and purification of recombinant *E. coli* HUα **(A)**, HUβ **(B)**, HUβ(K67ac) **(C)**, and HUβ(K86ac) **(D)** from *E. coli* BL21-AI. See section “Materials and Methods” for the detailed procedure. In each panel, expression and nickel affinity purification results are shown in (i) with lane 1 = whole cell lysate before protein induction, lane 2 = whole cell lysate after protein induction for 16 h, lane 3 = insoluble fraction of cell lysate, lane 4 = soluble fraction of cell lysate, lane 5 = flow through from nickel affinity column, lane 6 = flow through from column wash fraction 1, lane 7 = flow through from column wash fraction 2, lane 8 = eluted protein fraction 1, lane 9 = eluted protein fraction 2, lane 10 = eluted protein fraction 3, lane 11 = eluted protein fraction 4. For (ii), purity of four protein fractions eluted from cation exchange chromatography using a NaCl gradient and absorption at 214 nm for detection was analyzed by SDS-PAGE. Results of the pooled purified protein for further studies analyzed by SDS-PAGE and mass spectrometry are shown in (iii) and (iv), respectively. Molecular weights are shown in Dalton (Da), and theoretical values for HUα, HUβ, HUβ(K67ac), and HUβ(K86ac) are 10,357.5, 10,290.7, 10,332.7, and 10,332.7 Da, respectively. Deconvoluted ESI MS spectra are shown in the range of 5–15 kDa. The full-size spectra can be found in Supplementary Figure 1.

To produce HUα, a plasmid containing *hupA* was constructed through PCR amplification of the genomic DNA of *E. coli* BL21(DE3), followed by Gibson assembly into a pBAD expression vector. A C-terminal 6xHis tag was appended at the same time to facilitate protein purification. The resulting construct, pBAD hupA-His6, was confirmed by sanger sequencing and transformed into *E. coli* BL21 AI for protein expression (Figure 2A). The expression cultures were purified by affinity and cation-exchange chromatography. The identity of the purified product was confirmed by mass spectrometry (Figure 2A and Supplementary Figure 1), where the observed peak (10,357.5 Da) is in close agreement with the calculated molecular weight (10,357.8 Da). Following the same protocol, *E. coli* HUβ was expressed and purified (Figure 2B).

To produce the acetylated HUβ, site-directed mutagenesis was used to mutate the codon corresponding to Lys67 or Lys86 to the amber codon (TAG) in pBAD HupB-His6, resulting in plasmids pBAD hupB(K67TAG)-His6 and pBAD hupB(K86TAG)-His6, respectively. Each construct was co-transformed with plasmid pCDF AcKST (Liao et al., 2017), which expresses an orthogonal aminoacyl-tRNA synthetase/tRNA_*CUA*_ pair (Neumann et al., 2009) for acetyl lysine incorporation in response to the amber codon, into *E. coli* BL21 AI. During protein expression, the media was supplemented with 5 mM acetyl lysine and 20 mM nicotinamide, a deacetylase inhibitor. Following the same purification procedure, HUβ(K67ac) and HUβ(K86ac) were obtained and confirmed by mass spectrometry (Figures 2C,D).

### Effects of Acetylation on Binding to DNA

We performed the electrophoretic mobility shift assay to analyze DNA-binding capability of different *E. coli* HU dimers. In this assay, the protein is incubated with a DNA fragment. The mixture is then analyzed by electrophoresis to separate free DNA from DNA-protein complexes, which have reduced mobility in the gel.

**FIGURE 3:**
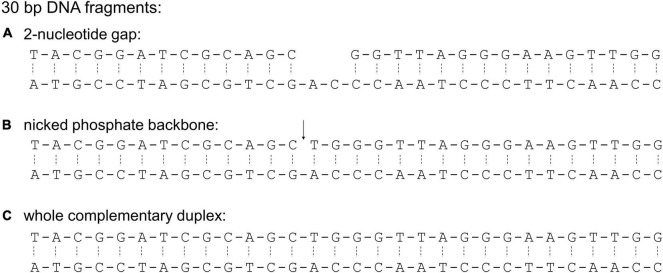
Sequences of 30-bp DNA fragments used in this study. These sequences were used to characterize binding preference of *E. coli* HU (Castaing et al., 1995). The sequences differ in the presence of a 2-nucleotide gap **(A)**, a nicked phosphate backbone (**B**, indicated by ↓), and a fully complementary sequence **(C)**. The phosphate backbone is indicated with a solid line while hydrogen bonds between complementary base pairs are indicated with a dashed line.

**FIGURE 4:**
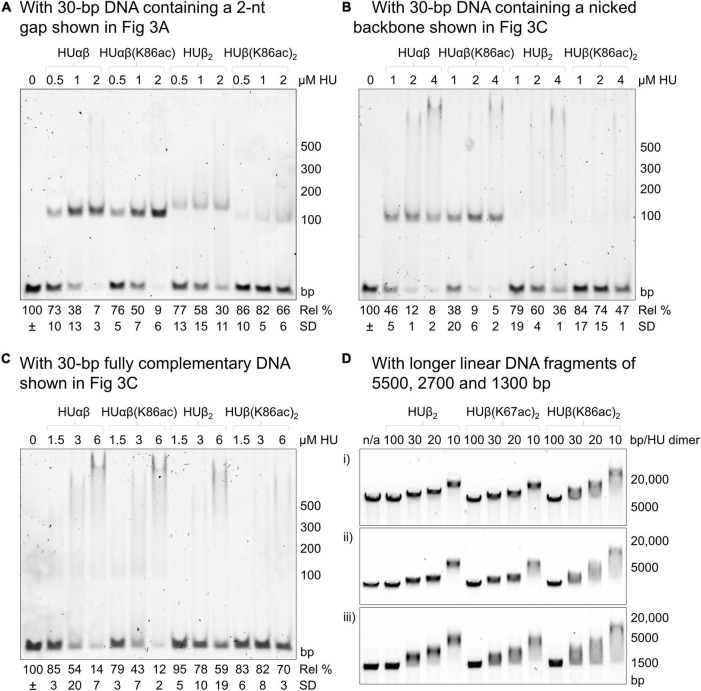
Effect of *E. coli* HUβ acetylation at Lys67 or Lys86 on the interaction of HU homodimer or heterodimer. Electrophoretic mobility shift assays were performed using either 30 bp DNA fragments containing a 2-nt gap **(A)**, nicked phosphate backbone **(B)**, or fully complementary sequence **(C)** or longer DNA fragments of 1300, 2700, or 5500 bp. The first lane contains only the DNA fragment in the binding buffer without any protein. The subsequent lanes contain the DNA fragment in the binding buffer with the indicated amount of HU dimer. For panels **(A–C)**, the migrated band around the 100 bp marker corresponds to the complexes of 1 HU dimer and 1 DNA molecule, while the band at the top of the gel corresponds to the complexes of 2 HU dimers and 1 DNA molecule. Free DNA remaining in HU-containing conditions was calculated relative to the band intensity of the DNA-only lane (=100%). Average free DNA remaining (Rel%) and standard deviation (SD) from three independent repeats are presented below a representative image. Images of the other two repeats are shown in Supplementary Figure 3. For panel **(D)**, HU concentrations are shown as the ratios of HU dimers per number of base pairs (1 HU dimer every 100, 30, 20, or 10 bp). A representative image from a minimum of three independent repeats is shown. Images of the other repeats are shown in Supplementary Figure 5. Sequences of the longer DNA fragments are shown in the Supplementary Material.

We used a 30-bp double-stranded DNA fragment containing a 2-nt gap as the model substrate (Figure 3A). This DNA fragment was previously demonstrated to be a preferred substrate of *E. coli* HU over a fully complementary double-stranded DNA molecule (Castaing et al., 1995). In the assays, 1 μM of the DNA fragment was incubated with different concentrations of HU dimers. Free DNA and HU-DNA complexes were resolved in polyacrylamide gel electrophoresis (Figure 4). We attributed the band migrating around the 100-bp DNA marker as a 1:1 HU dimer and DNA complex. The DNA-binding affinity was characterized by the amount of free, unbound DNA remaining in each condition. This was calculated by setting the intensity of the DNA only sample as the reference to quantify the relative amount of free DNA in the reactions containing HU dimers.

Complexes of the 2-nt gap DNA with HUβ(K67ac)_2_ and HUβ(K86ac)_2_ migrated slightly faster than that of HUβ_2_, likely due to the presence of fewer positive charges in the acetylated proteins. While HUβ(K67ac)_2_ and HUβ_2_ displayed very similar DNA binding profiles (Supplementary Figure 2A), acetylation at Lys86 reduced the DNA binding affinity significantly (*p* < 0.05) as more than fourfold free DNA was observed in the presence of 4 μM HU dimer. As HUαβ heterodimer is the major species in exponential and stationary phases of *E. coli* (Claret and Rouviere-Yaniv, 1997), we also reconstituted heterodimers of HUαβ, HUαβ(K67ac), and HUαβ(K86ac). All heterodimers showed higher affinity to the 2-nt gap DNA (Supplementary Figure 2B) compared to the homodimer (Figure 4A and Supplementary Figure 3), in agreement with previous reports (Castaing et al., 1995; Pinson et al., 1999). Intriguingly, acetylation at neither Lys67 nor Lys86 of HUβ had a significant impact (*p* > 0.15) on DNA binding by the heterodimers.

To further investigate how acetylation at Lys86 of HUβ affects binding with different types of DNA, a 30-bp double-stranded DNA containing a nicked phosphate backbone (Figure 3B) or the intact, fully complementary fragment (Figure 3C) was used as the substrate in the electrophoretic mobility shift assay. For the nicked DNA, again, no significant difference was observed between HUαβ and HUαβ(K86ac), while HUβ(K86ac) had a lower affinity to the nicked DNA than that of HUβ_2_ (Figure 4B). For the fully complementary DNA, acetylation at Lys86 of HUβ seemed to have no effect on DNA binding by either the hetero- or homodimer (Figure 4C). A similar effect was observed when a second 30 bp fully complementary DNA fragment with an alternative sequence was tested (Supplementary Figure 4).

Previously, we used long double-stranded DNA fragments (>3000 bp) from restriction digestion of plasmids as the substrates in electrophoretic mobility shift assays to investigate the effects of acetylation at Lys13 of *A. baumannii* HU (Liao et al., 2017). Thus, we wondered if increasing the length of fully complementary DNA from 30 bp would make any difference, and linear DNA fragments around 1300, 2700, and 5500 bp were employed as the substrates in electrophoretic mobility shift assays and analyzed by agarose gel electrophoresis (Figure 4D and Supplementary Figure 5). Again, the mobility profiles of HUβ_2_ and HUβ(K67ac)_2_ were similar, whereas more smeared bands were observed with samples incubated with HUβ(K86ac)_2_. Surprisingly, the most mobile end of the smear was in line with free DNA, and the band extended past the most mobility-restricted DNA in the HUβ_2_- or HUβ(K67ac)_2_-containing samples. A similar effect was observed when the HU variants were incubated with a 420 bp DNA fragment (Supplementary Figure 4C). These data suggest a greater level of retardation of the long linear DNA fragments by HUβ(K86ac)_2_ compared to the other two homodimer variants.

HUα_2_ and HUαβ have been reported to facilitate supercoiling of plasmid DNA in the presence of topoisomerase I (Broyles and Pettijohn, 1986; Bensaid et al., 1996; Kar et al., 2006; Guo and Adhya, 2007; Huang et al., 2021). Thus, we investigated whether acetylation at Lys86 influences DNA supercoiling capability of HUβ_2_ (Supplementary Figure 6). Incubation of relaxed plasmid DNA with HUβ_2_ and topoisomerase I resulted in the formation of intermediate partially supercoiled topoisomers. For HUβ(K86ac)_2_, a smear without any clear or defined bands was observed on the agarose gel and migrated much faster than the relaxed DNA. In fact, the smear extended from in line with the non-relaxed (supercoiled) plasmid to the end of the gel. It is difficult to judge whether HUβ(K86ac)_2_ can facilitate DNA supercoiling and to rationalize the experimental observation. Nevertheless, the results indicate that Lys86 acetylation affects how HUβ interacts with DNA.

### Effects of K86ac on Protein Secondary Structure and Thermal Stability

**FIGURE 5:**
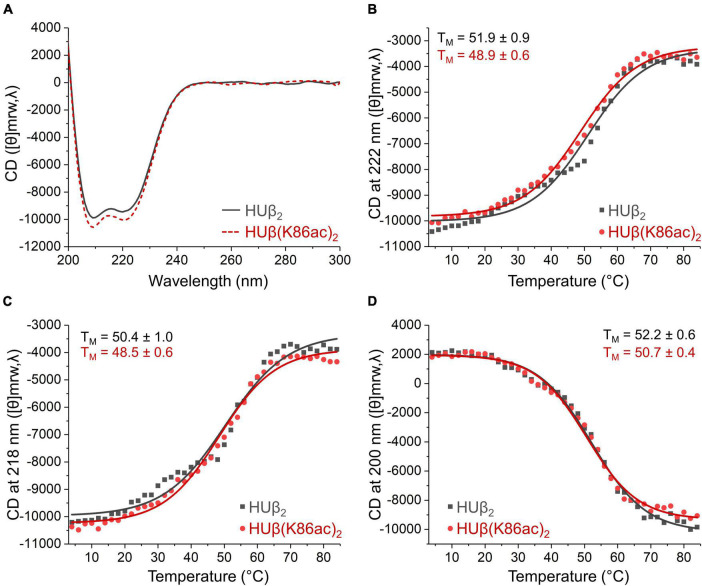
Effect of acetylation at Lys86 of *E. coli* HUβ_2_ homodimer on secondary structure and thermal stability analyzed by circular dichroism. Data of wild-type and acetylated dimers are shown in gray or red lines, respectively. **(A)** CD spectra from 200 to 300 nm measured at 20°C showing the characteristic α-helix signals with two negative bands of similar magnitude around 210 and 220 nm. **(B–D)** Melting temperature (T_*M*_) of the protein determined by CD signal from 4 to 84°C at 222 **(B)**, 218 **(C)**, and 200 **(D)** nm.

To investigate if Lys86 acetylation modulates DNA binding through a change in protein secondary structure or thermal stability, characterization of these physical properties was carried out using circular dichroism (CD). At 20°C, there were negligible differences in the CD spectra, indicating the preservation of the α-helix secondary structure (Figure 5A). Additionally, by measuring CD spectra across increasing temperatures, we were able to calculate the melting temperature (Figures 5B–D) and found no significant difference between HUβ_2_ and HUβ(K86ac)_2_. Further, analytical size exclusion chromatography confirmed that HU protein in all samples were in the dimeric form and acetylation did not influence dimer formation (Supplementary Figure 7).

## Discussion

HU has varying roles in bacterial gene transcription and virulence across bacterial species (Oberto et al., 2009; Mangan et al., 2011; Stojkova et al., 2018). Recently, HU has also been implicated in epigenetic regulation. Lys acetylation and methylation of *Mycobacterium tuberculosis* HU (encoded by *hupB*) have been demonstrated to heritably modulate bacterial transcription, growth and drug resistance, in a manner analogous to epigenetic regulation of eukaryotic histones (Sakatos et al., 2018). Specifically, post-translational modification of Lys86 was implicated in particular cell phenotypes, while K86R mutation led to about fourfold reduction in the number of colonies formed in the presence of an antibiotic. Thus, it is reasonable to hypothesize that site-specific acetylation of HU from other bacteria may act as epigenetic regulators in the corresponding species.

Of the five *E. coli* HUβ Lys residues subjected to acetylation *in vivo* (Castaño-Cerezo et al., 2014), Lys67 and Lys86 were chosen due to their locations on the DNA binding β-arms and DNA binding interface, respectively (Figure 1). Our results showed that HUβ(K86ac)_2_ displayed a significant reduction in affinity to 30-bp DNA containing a 2-nt gap or nick, while HUβ(K67ac)_2_ showed similar binding profiles to that of HUβ_2_ in all tested DNA fragments (Figure 4). It is noteworthy that the band corresponding to the complex of one HU dimer and one DNA molecule was not observed in the assays using the fully complementary 30-bp DNA (Figure 4C). Instead, as the amount of free DNA remaining decreases, smearing at higher molecular weight developed, in contrast to assays using 2-nt gap or nicked DNA fragments. This discrepancy may be due to alternative binding modes of HU depending on the type of DNA, although further investigations are needed to verify this hypothesis.

In agreement with the literature (Pinson et al., 1999), *E. coli* HUαβ heterodimer showed a significantly greater affinity for double-stranded DNA in comparison to HUβ_2_ homodimer (Figure 4 and Supplementary Figures 2–3). Interestingly, acetylation at either Lys67 or Lys86 of HUβ had negligible impact on the binding capacity of the heterodimer. Since both protein units were acetylated in the homodimers, whereas only one unit was acetylated in the heterodimers, the effect of acetylation on DNA binding may not be as prominent in the heterodimers. Furthermore, the different types of dimers have been reported to interact differently with DNA (Pinson et al., 1999), which may also account for the lack of obvious effect in the heterodimer. Additionally, while acetylation of other lysine residues was identified in cells isolated during both the exponential and stationary phases, acetylation at Lys86 of HUβ was only identified in the stationary phase. Similarly, acetylation at Lys83 of HUα was only identified during the exponential phase, whereas acetylation at position 13/18/67/86 of HUα were found in both exponential and stationary phases (Castaño-Cerezo et al., 2014). This may indicate that modification of these residues has specific roles corresponding to the homodimers, as HUα_2_ is more likely to be formed in the exponential phase and HUβ_2_ in the stationary phase. We regret that we were unable to investigate the effect of acetylation on HUα, both as a subunit of the heterodimer and in the homodimer. Further research to determine if DNA binding capacity is reduced, as it is in HUβ_2_, will be critical for determining the extent of acetylation as a mechanism for modulating the DNA binding activity of HU *in vivo*, as HUα_2_ predominates in the exponential phase and HUαβ accounts for the majority of HU dimers in the stationary phase (Claret and Rouviere-Yaniv, 1997).

In assays with longer double-stranded DNA molecules (i.e., 1300, 3800, or 5500 bp), DNA incubated with HUβ(K86ac)_2_ consistently displayed decreased migration in the agarose gels and increased smearing, suggesting more HU dimers were bound to the DNA. Increased smearing may also highlight a difference in DNA bending or conformation, resulting in less uniformity of DNA fragments in the reactions and a more smeared band appearance. One plausible explanation for the stronger binding by HUβ(K86ac)_2_ is that long DNA molecules may have different interactions with HUβ homodimers compared to 30-bp DNA fragments; it has been documented that interaction of HU with DNA varies depending on DNA length (Swinger et al., 2003; Hammel et al., 2016). Alternatively, long DNA molecules may have local secondary structures favored by HUβ(K86ac)_2_.

Overall, acetylation at Lys67 of HUβ did not seem to affect DNA binding. The results were initially surprising as the residue is located in the β-arms that interact directly with DNA. However, Lys67 is not highly conserved (Grove and Saavedra, 2002; Kamashev et al., 2017); the negatively charged glutamic acid is often present at this position, suggesting that alteration to the charge of this side chain may not be critical to protein function. In contrast, Lys86 is highly conserved and has been reported as a key residue involved in HU-DNA interactions across bacterial species (Grove and Saavedra, 2002; Bhowmick et al., 2014; Kamashev et al., 2017). Our results bolster this assertion while also demonstrating that acetylation of this residue may be used to modulate DNA-binding capacity.

Neutralization of the positive charge at Lys86 can also be achieved by amino acid substitution. Indeed, replacement of Lys86 with alanine reduced the DNA binding capability of *E. coli* HUαβ (Thakur et al., 2021). A similar effect was found with *Bacillus subtilis* HU, where K86A mutation reduced the DNA binding capacity to 20% of the wild-type protein (Köhler and Marahiel, 1998). In a HU homolog, K86Q also reduced the DNA binding affinity (Grove and Saavedra, 2002). Lys86, among other residues, was also a target in the design of an effective inhibitor, which prevented interaction with DNA and reduced binding affinity of *M. tuberculosis* HU (Bhowmick et al., 2014).

*In vivo* multiple residues may be modified simultaneously. Therefore, Lys67 and Lys86 may be acetylated at the same time, giving a compounded effect on protein properties. Indeed, Thakur et al. (2021) investigated modifications of *E. coli* HUβ and found when either Lys83 or Lys86 was replaced with alanine, or when the DNA-binding β-arms were deleted, HU could still bind DNA albeit with reduced affinity. However, when one residue was replaced with alanine and the DNA binding loop was deleted, no DNA binding capacity remained (Thakur et al., 2021). In this case, compromise of either site was tolerated but simultaneous modification of both sites rendered HU unable to bind DNA. Hence, it is possible that acetylation of both Lys67 and Lys86 may have a more pronounced effect on HU-DNA interaction.

HU is a truly versatile protein serving many different and sometimes contrasting functions in cells (Dame and Goosen, 2002; Grove, 2011; Stojkova et al., 2019). HU exhibits a minimum of two binding modes (Swinger et al., 2003; Koh et al., 2011; Hammel et al., 2016). For short, structurally deformed DNA fragments, HU binds the DNA with its arms and bends the DNA around its body. For native DNA, HU mainly interacts through its α-helical body and pairs of dimers interact across the DNA strand. Thus, it seems possible that the effect of acetylation may also have contrasting consequences depending on the type of DNA being interacted with and may therefore influence cellular processes in different ways. For example, acetylation of the protein could also be a mechanism of controlling the local concentration of HU in the cell. Deacetylating HU protein would increase the concentration in the cytosol by decreasing affinity for genomic and plasmid DNA, making more HU available to bind DNA damage repair intermediates. Clearly, considerably more work is needed to unveil the molecular basis of the varying interactions of HU with different types of DNA.

This work has provided a glimpse into how site-specific post-translational modification of *E. coli* HUβ may regulate protein function. We have also demonstrated how genetic code expansion can be useful for studying site-specific post-translational modification (Chen and Tsai, 2021). There is of course considerably more to be done to uncover the effect of post-translational modification of HU from other bacterial species and to determine whether these modifications are involved in transcriptional regulation to modulate bacterial phenotypes, such as growth, pathogenicity and drug resistance. The *in vivo* effects of site-specific lysine acetylation can theoretically be studied through genetic incorporation of non-hydrolyzable acetyl lysine analogs (Venkat et al., 2017; Zhang et al., 2018) although we have had very limited success in this approach. Alternatively, one can perform site-specific incorporation of acetyl lysine in deacetylase-knockout strains to obtain stoichiometrically acetylated proteins *in vivo* for functional studies. Unraveling the biological functions of molecular modifications deepens our understanding of the delicate controls regulating cellular physiology and can potentially uncover new antimicrobial targets.

## Data Availability Statement

The original contributions presented in the study are included in the article/Supplementary Material. Original files of CD data have been deposited to the Cardiff University data catalog at http://doi.org/10.17035/d.2021.0143930703. Further inquiries can be directed to the corresponding author.

## Author Contributions

VB and Y-HT: conceptualization, formal analysis, methodology, and manuscript preparation. VB: data curation and investigation. Y-HT: funding acquisition, project administration, resources, and supervision. Both authors contributed to the article and approved the submitted version.

## Conflict of Interest

The authors declare that the research was conducted in the absence of any commercial or financial relationships that could be construed as a potential conflict of interest.

## Publisher’s Note

All claims expressed in this article are solely those of the authors and do not necessarily represent those of their affiliated organizations, or those of the publisher, the editors and the reviewers. Any product that may be evaluated in this article, or claim that may be made by its manufacturer, is not guaranteed or endorsed by the publisher.
