# Supplementary material for: Acetylation at Lysine 86 of Escherichia coli HUβ Modulates the DNA-Binding Capability of the Protein
Source: Front Microbiol. 2022 Feb 4;12:809030. doi: 10.3389/fmicb.2021.809030 (PMC8854993; doi:10.3389/fmicb.2021.809030)
Supplement: Supplementary file 1 [file Data_Sheet_1.pdf]

frontiers

Supplementary Material

1 Supplementary Data

1.1 DNA sequences used in EMSA.

Sticky ends (single stranded DNA) are annotated in red.

5500 bp DNA sequence - pCX sfGFP(150TAA) linearised with HindIII digestion:

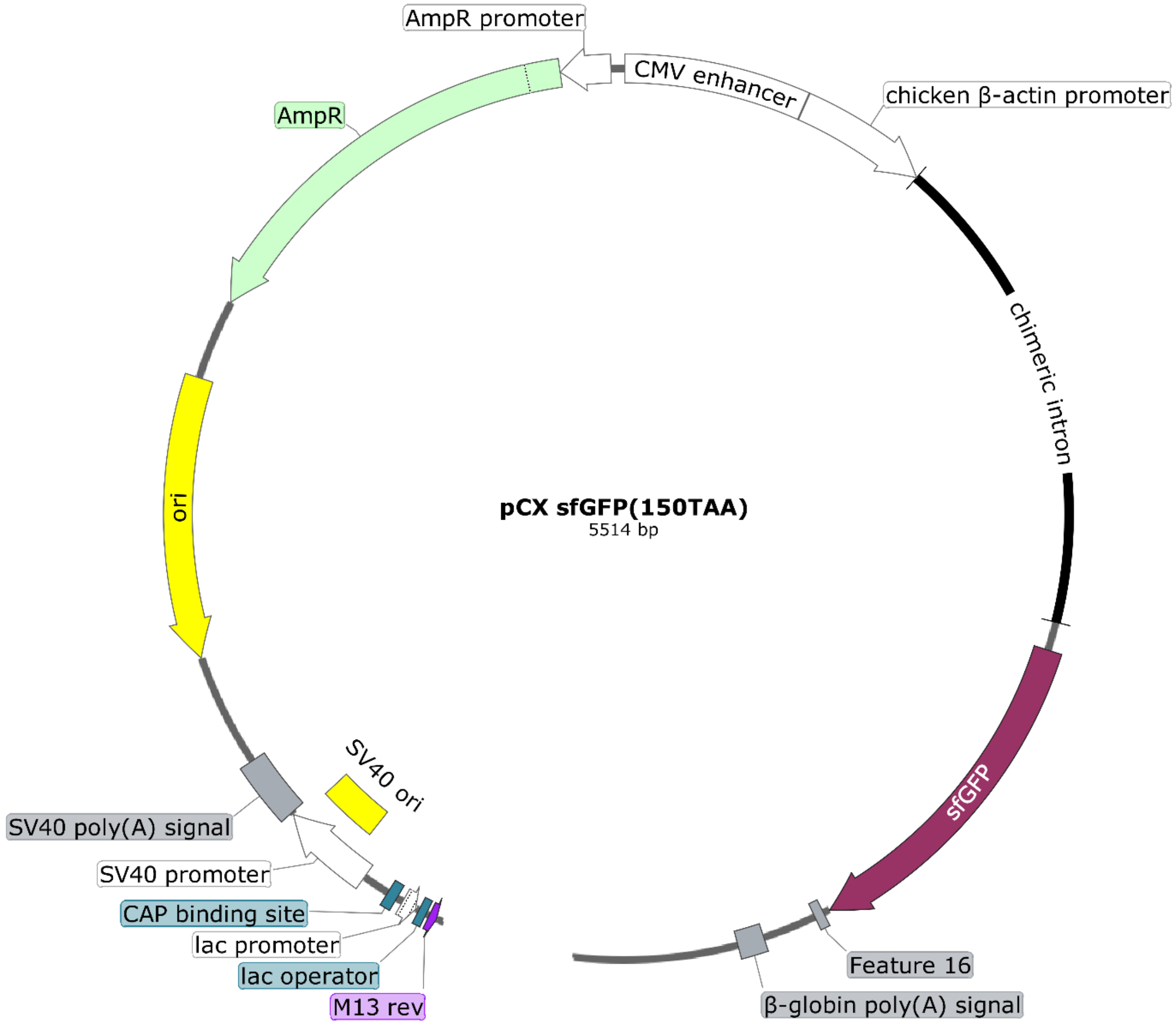

AGCTTGGCGTAATCATGGTCATAGCTGTTTCCTGTGTGAAATTGTTATCCGCTCACAATT
CCACACAACATACGAGCCGGAAGCATAAAGTGTAAAGCCTGGGGTGCCTAATGAGTGA
GCTAACTCACATTAATTGCGTTGCGCTCACTGCCCGCTTTCCAGTCGGGAAACCTGTCGT
GCCAGCGGATCCGCATCTCAATTAGTCAGCAACCATAGTCCCGCCCCTAACTCCGCCCAT
CCCGCCCCTAACTCCGCCCAGTTCCGCCCATTCTCCGCCCCATGGCTGACTAATTTTTTTT

ATTTATGCAGAGGCCGAGGCCGCCTCGGCCTCTGAGCTATTCCAGAAGTAGTGAGGAGG
CTTTTTTGGAGGCCTAGGCTTTTGCAAAAAGCTAACTTGTTTATTGCAGCTTATAATGGT
TACAAATAAAGCAATAGCATCACAAATTTCACAAATAAAGCATTTTTTTCACTGCATTCT
AGTTGTGGTTTGTCCAAACTCATCAATGTATCTTATCATGTCTGGATCCGCTGCATTAAT
GAATCGGCCAACGCGCGGGGAGAGGCGGTTTGCGTATTGGGCGCTCTTCCGCTTCCTCG
CTCACTGACTCGCTGCGCTCGGTCGTTCGGCTGCGGCGAGCGGTATCAGCTCACTCAAA
GGCGGTAATACGGTTATCCACAGAATCAGGGGATAACGCAGGAAAGAACATGTGAGCA
AAAGGCCAGCAAAAGGCCAGGAACCGTAAAAAGGCCGCGTTGCTGGCGTTTTTCCATAG
GCTCCGCCCCCCTGACGAGCATCACAAAAATCGACGCTCAAGTCAGAGGTGGCGAAACC
CGACAGGACTATAAAGATACCAGGCGTTTCCCCCTGGAAGCTCCCTCGTGCGCTCTCCTG
TTCCGACCCTGCCGCTTACCGGATACCTGTCCGCCTTTCTCCCTTCGGGAAGCGTGGCGC
TTTCTCAATGCTCACGCTGTAGGTATCTCAGTTCGGTGTAGGTCGTTCGCTCCAAGCTGG
GCTGTGTGCACGAACCCCCCGTTCAGCCCGACCGCTGCGCCTTATCCGGTAACTATCGTC
TTGAGTCCAACCCGGTAAGACACGACTTATCGCCACTGGCAGCAGCCACTGGTAACAGG
ATTAGCAGAGCGAGGTATGTAGGCGGTGCTACAGAGTTCTTGAAGTGGTGGCCTAACTA
CGGCTACACTAGAAGGACAGTATTTGGTATCTGCGCTCTGCTGAAGCCAGTTACCTTCGG
AAAAAGAGTTGGTAGCTCTTGATCCGGCAAACAAACCACCGCTGGTAGCGGTGGTTTTT
TTGTTTGCAAGCAGCAGATTACGCGCAGAAAAAAAGGATCTCAAGAAGATCCTTTGATC
TTTTCTACGGGGTCTGACGCTCAGTGGAACGAAAACTCACGTTAAGGGATTTTGGTCATG
AGATTATCAAAAAGGATCTTCACCTAGATCCTTTTAAATTAAAAATGAAGTTTTAAATCA
ATCTAAAGTATATATGAGTAAACTTGGTCTGACAGTTACCAATGCTTAATCAGTGAGGC
ACCTATCTCAGCGATCTGTCTATTTCGTTCATCCATAGTTGCCTGACTCCCCGTCGTGTAG
ATAACTACGATACGGGAGGGCTTACCATCTGGCCCCAGTGCTGCAATGATACCGCGAGA
CCCACGCTCACCGGCTCCAGATTTATCAGCAATAAACCAGCCAGCCGGAAGGGCCGAGC
GCAGAAGTGGTCCTGCAACTTTATCCGCCTCCATCCAGTCTATTAATTGTTGCCGGGAAG
CTAGAGTAAGTAGTTCGCCAGTTAATAGTTTGCGCAACGTTGTTGCCATTGCTACAGGCA
TCGTGGTGTCACGCTCGTCGTTTGGTATGGCTTCATTCAGCTCCGGTTCCCAACGATCAA
GGCGAGTTACATGATCCCCCATGTTGTGCAAAAAAGCGGTTAGCTCCTTCGGTCCTCCGA
TCGTTGTCAGAAGTAAGTTGGCCGCAGTGTTATCACTCATGGTTATGGCAGCACTGCATA
ATTCTCTTACTGTCATGCCATCCGTAAGATGCTTTTCTGTGACTGGTGAGTACTCAACCA
AGTCATTCTGAGAATAGTGTATGCGGCGACCGAGTTGCTCTTGCCCGGCGTCAATACGG
GATAATACCGCGCCACATAGCAGAACTTTAAAAGTGCTCATCATTGGAAAACGTTCTTC
GGGGCGAAAACTCTCAAGGATCTTACCGCTGTTGAGATCCAGTTCGATGTAACCCACTC
GTGCACCCAACTGATCTTCAGCATCTTTTACTTTCACCAGCGTTTCTGGGTGAGCAAAAA
CAGGAAGGCAAAATGCCGCAAAAAAGGGAATAAGGGCGACACGGAAATGTTGAATACT
CATACTCTTCCTTTTTCAATATTATTGAAGCATTTATCAGGGTTATTGTCTCATGAGCGGA
TACATATTTGAATGTATTTAGAAAAATAAACAAATAGGGGTTCCGCGCACATTTCCCCG
AAAAGTGCCACCTGGTCGACATTGATTATTGACTAGTTATTAATAGTAATCAATTACGGG
GTCATTAGTTCATAGCCCATATATGGAGTTCCGCGTTACATAACTTACGGTAAATGGCCC
GCCTGGCTGACCGCCCAACGACCCCCGCCCATTGACGTCAATAATGACGTATGTTCCCAT
AGTAACGCCAATAGGGACTTTCCATTGACGTCAATGGGTGGACTATTTACGGTAAACTG
CCCACTTGGCAGTACATCAAGTGTATCATATGCCAAGTACGCCCCCTATTGACGTCAATG
ACGGTAAATGGCCCGCCTGGCATTATGCCCAGTACATGACCTTATGGGACTTTCCTACTT
GGCAGTACATCTACGTATTAGTCATCGCTATTACCATGGGTCGAGGTGAGCCCCACGTTC
TGCTTCACTCTCCCCATCTCCCCCCCCTCCCCACCCCCAATTTTGTATTTATTTATTTTTTA
ATTATTTTGTGCAGCGATGGGGGCGGGGGGGGGGGGGGCGCGCGCCAGGCGGGGCGGG
GCGGGGCGAGGGGCGGGGCGGGGCGAGGCGGAGAGGTGCGGCGGCAGCCAATCAGAG

CGGCGCGCTCCGAAAGTTTCCTTTTATGGCGAGGCGGCGGCGGCGGCGGCCCTATAAAA
AGCGAAGCGCGCGGCGGGCGGGAGTCGCTGCGTTGCCTTCGCCCCGTGCCCCGCTCCGC
GCCGCCTCGCGCCGCCCGCCCCGGCTCTGACTGACCGCGTTACTCCCACAGGTGAGCGG
GCGGGACGGCCCTTCTCCTCCGGGCTGTAATTAGCGCTTGGTTTAATGACGGCTCGTTTC
TTTTCTGTGGCTGCGTGAAAGCCTTAAAGGGCTCCGGGAGGGCCCTTTGTGCGGGGGGG
AGCGGCTCGGGGGGTGCGTGCGTGTGTGTGTGCGTGGGGAGCGCCGCGTGCGGCCCGCG
CTGCCCGGCGGCTGTGAGCGCTGCGGGCGCGGCGCGGGGCTTTGTGCGCTCCGCGTGTG
CGCGAGGGGAGCGCGGCCGGGGGCGGTGCCCCGCGGTGCGGGGGGGCTGCGAGGGGAA
CAAAGGCTGCGTGCGGGGTGTGTGCGTGGGGGGGTGAGCAGGGGGTGTGGGCGCGGCG
GTCGGGCTGTAACCCCCCCCTGCACCCCCCTCCCCGAGTTGCTGAGCACGGCCCGGCTTC
GGGTGCGGGGCTCCGTACGGGGCGTGGCGCGGGGCTCGCCGTGCCGGGCGGGGGGTGG
CGGCAGGTGGGGGTGCCGGGCGGGGCGGGGCCGCCTCGGGCCGGGGAGGGCTCGGGGG
AGGGGCGCGGCGGCCCCCGGAGCGCCGGCGGCTGTCGAGGCGCGGCGAGCCGCAGCCA
TTGCCTTTTATGGTAATCGTGCGAGAGGGCGCAGGGACTTCCTTTGTCCCAAATCTGTGC
GGAGCCGAAATCTGGGAGGCGCCGCCGCACCCCCTCTAGCGGGCGCGGGGCGAAGCGG
TGCGGCGCCGGCAGGAAGGAAATGGGCGGGGAGGGCCTTCGTGCGTCGCCGCGCCGCC
GTCCCCTTCTCCCTCTCCAGCCTCGGGGCTGTCCGCGGGGGGACGGCTGCCTTCGGGGGG
GACGGGGCAGGGCGGGGTTCGGCTTCTGGCGTGTGACCGGCGGCTCTAGAGCCTCTGCT
AACCATGTTCATGCCTTCTTCTTTTTCCTACAGCTCCTGGGCAACGTGCTGGTTGTTGTGC
TGTCTCATCATTTTGGCAAAGAATTCGCCACCATGGTGAGCAAGGGCGAGGAGCTGTTC
ACCGGCGTGGTGCCCATCCTGGTGGAGCTGGACGGCGACGTGAACGGCCACAAGTTCAG
CGTGCGCGGCGAGGGCGAGGGCGACGCCACCAACGGCAAGCTGACCCTGAAGTTCATC
TGCACCACCGGCAAGCTGCCCGTGCCCTGGCCCACCCTGGTGACCACCCTGACCTACGG
CGTGCAGTGCTTCAGCCGCTACCCCGACCACATGAAGCGCCACGACTTCTTCAAGAGCG
CCATGCCCGAGGGCTACGTGCAGGAGCGCACCATCAGCTTCAAGGACGACGGCACCTAC
AAGACCCGCGCCGAGGTGAAGTTCGAGGGCGACACCCTGGTGAACCGCATCGAGCTGA
AGGGCATCGACTTCAAGGAGGACGGCAACATCCTGGGCCACAAGCTGGAGTACAACTTC
AACAGCCACTAAGTGTACATCACCGCCGACAAGCAGAAGAACGGCATCAAGGCCAACT
TCAAGATCCGCCACAACGTGGAGGACGGCAGCGTGCAGCTGGCCGACCACTACCAGCA
GAACACCCCCATCGGCGACGGCCCCGTGCTGCTGCCCGACAACCACTACCTGAGCACCC
AGAGCGTGCTGAGCAAGGACCCCAACGAGAAGCGCGACCACATGGTGCTGCTGGAGTT
CGTGACCGCCGCCGGCATCACCCACGGCATGGACGAGCTGTACAAGTGAGAATTCACTC
CTCAGGTGCAGGCTGCCTATCAGAAGGTGGTGGCTGGTGTGGCCAATGCCCTGGCTCAC
AAATACCACTGAGATCTTTTTCCCTCTGCCAAAAATTATGGGGACATCATGAAGCCCCTT
GAGCATCTGACTTCTGGCTAATAAAGGAAATTTATTTTCATTGCAATAGTGTGTTGGAAT
TTTTTGTGTCTCTCACTCGGAAGGACATATGGGAGGGCAAATCATTTAAAACATCAGAA
TGAGTATTTGGTTTAGAGTTTGGCAACATATGCCATATGCTGGCTGCCATGAACAAAGGT
GGCTATAAAGAGGTCATCAGTATATGAAACAGCCCCCTGCTGTCCATTCCTTATTCCATA
GAAAAGCCTTGACTTGAGGTTAGATTTTTTTTATATTTTGTTTTGTGTTATTTTTTTCTTTA
ACATCCCTAAAATTTTCCTTACATGTTTTACTAGCCAGATTTTTCCTCCTCTCCTGACTAC
TCCCAGTCATAGCTGTCCCTCTTCTCTTATGAAGATCCCTCGACCTGCAGCCCAAGCT

2700 bp DNA sequence – pUC18 linearised with HindIII digestion:

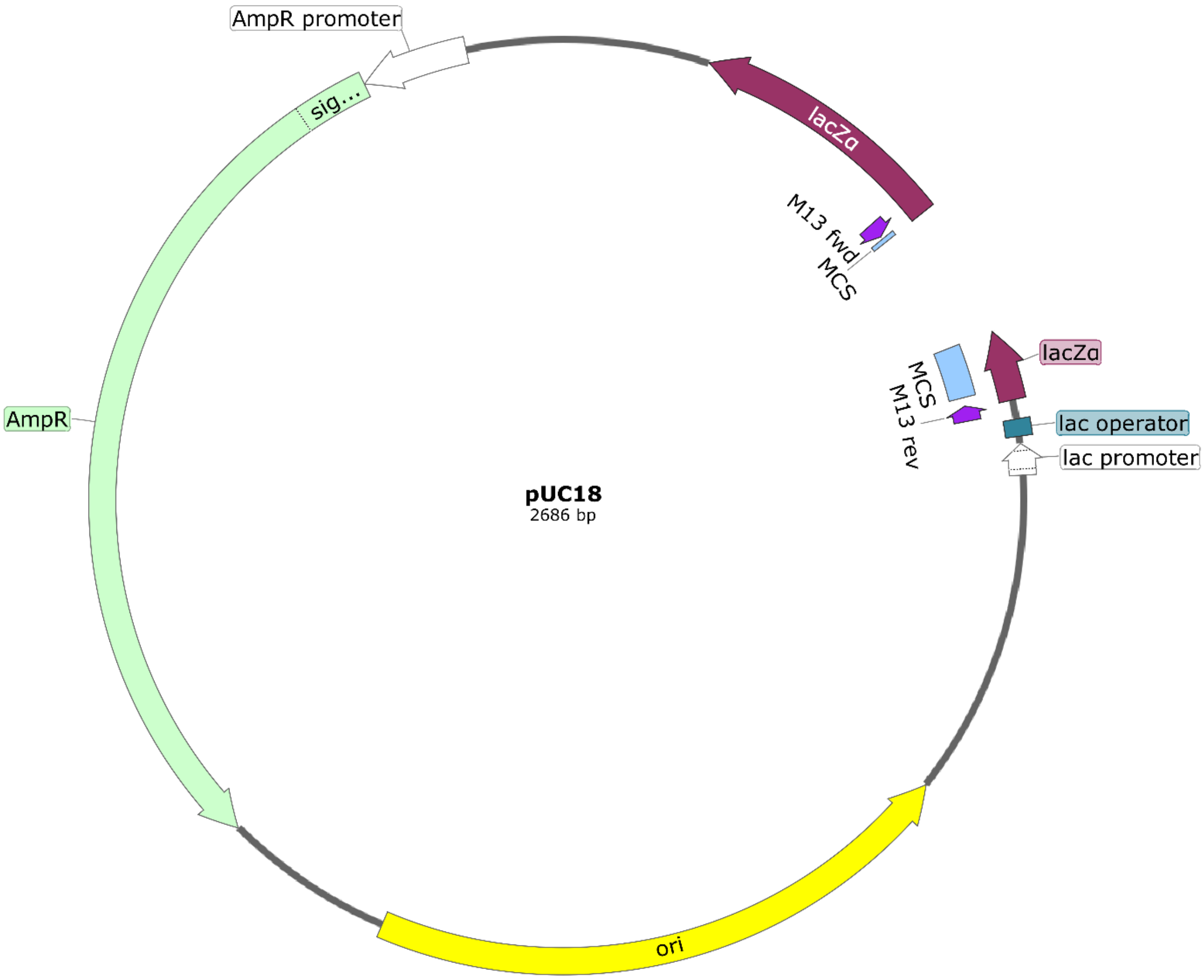

AGCTTGCATGCCTGCAGGTCGACTCTAGAGGATCCCCGGGTACCGAGCTCGAATTCGTA
ATCATGGTCATAGCTGTTTCCTGTGTGAAATTGTTATCCGCTCACAATTCCACACAACAT
ACGAGCCGGAAGCATAAAGTGTAAAGCCTGGGGTGCCTAATGAGTGAGCTAACTCACAT
TAATTGCGTTGCGCTCACTGCCCGCTTTCCAGTCGGGAAACCTGTCGTGCCAGCTGCATT
AATGAATCGGCCAACGCGCGGGGAGAGGCGGTTTGCGTATTGGGCGCTCTTCCGCTTCC
TCGCTCACTGACTCGCTGCGCTCGGTCGTTCGGCTGCGGCGAGCGGTATCAGCTCACTCA
AAGGCGGTAATACGGTTATCCACAGAATCAGGGGATAACGCAGGAAAGAACATGTGAG
CAAAAGGCCAGCAAAAGGCCAGGAACCGTAAAAAGGCCGCGTTGCTGGCGTTTTTCCAT
AGGCTCCGCCCCCCTGACGAGCATCACAAAAATCGACGCTCAAGTCAGAGGTGGCGAA
ACCCGACAGGACTATAAAGATACCAGGCGTTTCCCCCTGGAAGCTCCCTCGTGCGCTCT
CCTGTTCCGACCCTGCCGCTTACCGGATACCTGTCCGCCTTTCTCCCTTCGGGAAGCGTG
GCGCTTTCTCATAGCTCACGCTGTAGGTATCTCAGTTCGGTGTAGGTCGTTCGCTCCAAG
CTGGGCTGTGTGCACGAACCCCCCGTTCAGCCCGACCGCTGCGCCTTATCCGGTAACTAT
CGTCTTGAGTCCAACCCGGTAAGACACGACTTATCGCCACTGGCAGCAGCCACTGGTAA
CAGGATTAGCAGAGCGAGGTATGTAGGCGGTGCTACAGAGTTCTTGAAGTGGTGGCCTA
ACTACGGCTACACTAGAAGGACAGTATTTGGTATCTGCGCTCTGCTGAAGCCAGTTACCT

TCGGAAAAAGAGTTGGTAGCTCTTGATCCGGCAAACAAACCACCGCTGGTAGCGGTGGT
TTTTTTGTTTGCAAGCAGCAGATTACGCGCAGAAAAAAAGGATCTCAAGAAGATCCTTT
GATCTTTTCTACGGGGTCTGACGCTCAGTGGAACGAAAACTCACGTTAAGGGATTTTGGT
CATGAGATTATCAAAAAGGATCTTCACCTAGATCCTTTTAAATTAAAAATGAAGTTTTAA
ATCAATCTAAAGTATATATGAGTAAACTTGGTCTGACAGTTACCAATGCTTAATCAGTGA
GGCACCTATCTCAGCGATCTGTCTATTTCGTTCATCCATAGTTGCCTGACTCCCCGTCGTG
TAGATAACTACGATACGGGAGGGCTTACCATCTGGCCCCAGTGCTGCAATGATACCGCG
AGACCCACGCTCACCGGCTCCAGATTTATCAGCAATAAACCAGCCAGCCGGAAGGGCCG
AGCGCAGAAGTGGTCCTGCAACTTTATCCGCCTCCATCCAGTCTATTAATTGTTGCCGGG
AAGCTAGAGTAAGTAGTTCGCCAGTTAATAGTTTGCGCAACGTTGTTGCCATTGCTACAG
GCATCGTGGTGTCACGCTCGTCGTTTGGTATGGCTTCATTCAGCTCCGGTTCCCAACGAT
CAAGGCGAGTTACATGATCCCCCATGTTGTGCAAAAAAGCGGTTAGCTCCTTCGGTCCTC
CGATCGTTGTCAGAAGTAAGTTGGCCGCAGTGTTATCACTCATGGTTATGGCAGCACTGC
ATAATTCTCTTACTGTCATGCCATCCGTAAGATGCTTTTCTGTGACTGGTGAGTACTCAA
CCAAGTCATTCTGAGAATAGTGTATGCGGCGACCGAGTTGCTCTTGCCCGGCGTCAATA
CGGGATAATACCGCGCCACATAGCAGAACTTTAAAAGTGCTCATCATTGGAAAACGTTC
TTCGGGGCGAAAACTCTCAAGGATCTTACCGCTGTTGAGATCCAGTTCGATGTAACCCA
CTCGTGCACCCAACTGATCTTCAGCATCTTTTACTTTCACCAGCGTTTCTGGGTGAGCAA
AAACAGGAAGGCAAAATGCCGCAAAAAAGGGAATAAGGGCGACACGGAAATGTTGAA
TACTCATACTCTTCCTTTTTCAATATTATTGAAGCATTTATCAGGGTTATTGTCTCATGAG
CGGATACATATTTGAATGTATTTAGAAAAATAAACAAATAGGGGTTCCGCGCACATTTC
CCCGAAAAGTGCCACCTGACGTCTAAGAAACCATTATTATCATGACATTAACCTATAAA
AATAGGCGTATCACGAGGCCCTTTCGTCTCGCGCGTTTCGGTGATGACGGTGAAAACCT
CTGACACATGCAGCTCCCGGAGACGGTCACAGCTTGTCTGTAAGCGGATGCCGGGAGCA
GACAAGCCCGTCAGGGCGCGTCAGCGGGTGTTGGCGGGTGTCGGGGCTGGCTTAACTAT
GCGGCATCAGAGCAGATTGTACTGAGAGTGCACCATATGCGGTGTGAAATACCGCACAG
ATGCGTAAGGAGAAAATACCGCATCAGGCGCCATTCGCCATTCAGGCTGCGCAACTGTT
GGGAAGGGCGATCGGTGCGGGCCTCTTCGCTATTACGCCAGCTGGCGAAAGGGGGATGT
GCTGCAAGGCGATTAAGTTGGGTAACGCCAGGGTTTTCCCAGTCACGACGTTGTAAAAC
GACGGCCAGTGCCAAGCT

1300 bp PCR fragment – PCR of gene encoding maltose binding protein:

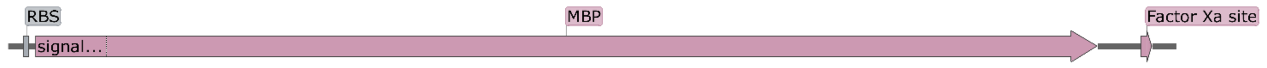

TTTTGTTTAACTTTAAGAAGGAGATATACATATGAAAATAAAAACAGGTGCACGCATCC
TCGCATTATCCGCATTAACGACGATGATGTTTTCCGCCTCGGCTCTCGCCAAAATCGAAG
AAGGTAAACTGGTAATCTGGATTAACGGCGATAAAGGCTATAACGGTCTCGCTGAAGTC
GGTAAGAAATTCGAGAAAGATACCGGAATTAAAGTCACCGTTGAGCATCCGGATAAACT
GGAAGAGAAATTCCCACAGGTTGCGGCAACTGGCGATGGCCCTGACATTATCTTCTGGG
CACACGACCGCTTTGGTGGCTACGCTCAATCTGGCCTGTTGGCTGAAATCACCCCGGAC
AAAGCGTTCCAGGACAAGCTGTATCCGTTTACCTGGGATGCCGTACGTTACAACGGCAA
GCTGATTGCTTACCCGATCGCTGTTGAAGCGTTATCGCTGATTTATAACAAAGATCTGCT
GCCGAACCCGCCAAAAACCTGGGAAGAGATCCCGGCGCTGGATAAAGAACTGAAAGCG

AAAGGTAAGAGCGCGCTGATGTTCAACCTGCAAGAACCGTACTTCACCTGGCCGCTGAT
TGCTGCTGACGGGGGTTATGCGTTCAAGTATGAAAACGGCAAGTACGACATTAAAGACG
TGGGCGTGGATAACGCTGGCGCGAAAGCGGGTCTGACCTTCCTGGTTGACCTGATTAAA
AACAAACACATGAATGCAGACACCGATTACTCCATCGCAGAAGCTGCCTTTAATAAAGG
CGAAACAGCGATGACCATCAACGGCCCGTGGGCATGGTCCAACATCGACACCAGCAAA
GTGAATTATGGTGTAACGGTACTGCCGACCTTCAAGGGTCAACCATCCAAACCGTTCGTT
GGCGTGCTGAGCGCAGGTATTAACGCCGCCAGTCCGAACAAAGAGCTGGCAAAAGAGT
TCCTCGAAAACTATCTGCTGACTGATGAAGGTCTGGAAGCGGTTAATAAAGACAAACCG
CTGGGTGCCGTAGCGCTGAAGTCTTACGAGGAAGAGTTGGTGAAAGATCCGCGTATTGC
CGCCACTATGGAAAACGCCCAGAAAGGTGAAATCATGCCGAACATCCCGCAGATGTCCG
CTTTCTGGTATGCCGTGCGTACTGCGGTGATCAACGCCGCCAGCGGTCGTCAGACTGTCG
ATGAAGCCCTGAAAGACGCGCAGACTAATTCGAGCTCGAACAACAACAACAATAACAA
TAACAACAACCTCGGGATCGAGGGAAGGGCTAGCGAGAATCTTTATTTTCAGG

2 Supplementary Figures and Tables

2.1 Supplementary Figures

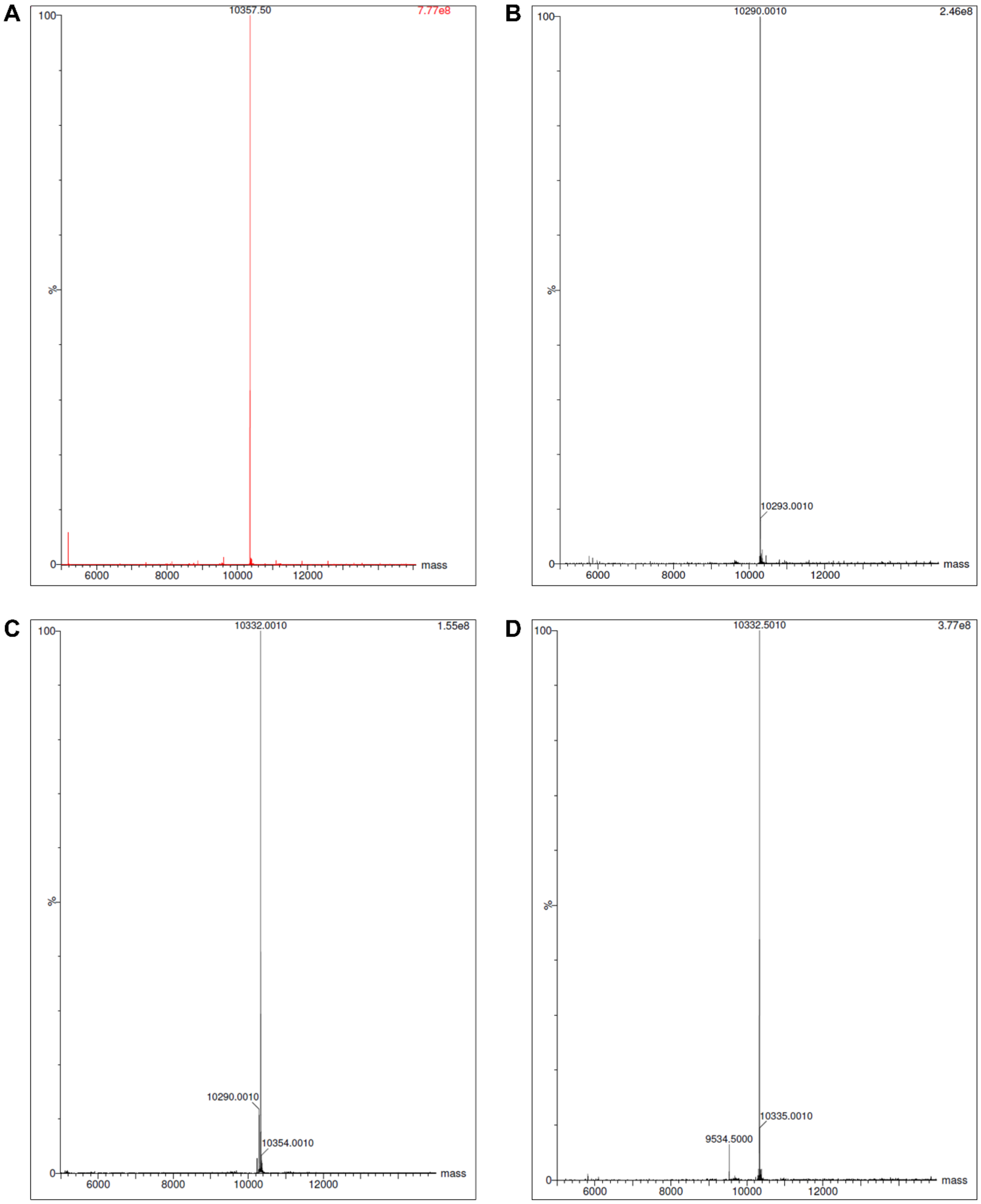

Supplementary Figure 1. Mass spectrometry analysis of purified proteins from **Figure 2** of the main manuscript. Mass spectrometry analysis of purified (**A**) HUα, (**B**) HUβ, (**C**) HUβ(K67ac) and (**D**) HUβ(K86ac).

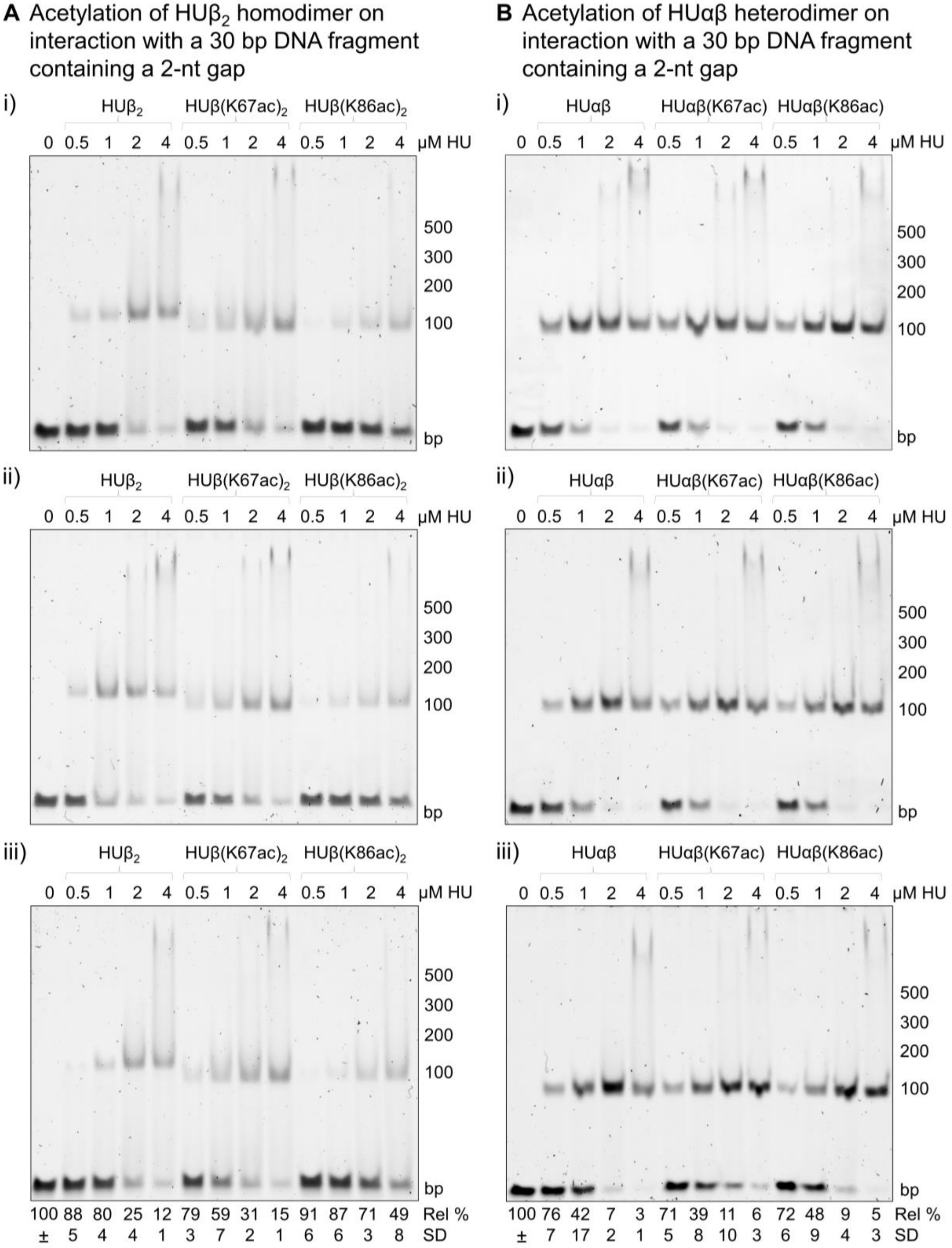

Supplementary Figure 2. Effect of acetylation of K67ac and K86ac on interaction with 30-bp DNA containing 2-nucleotide gap. EMSA of *E. coli* HUβ2 (**A**) or HUαβ (**B**) with 30-bp DNA containing a 2-nt gap (shown in **Figure 3A**). Free DNA remaining in HU-containing conditions was calculated relative to the band intensity of the DNA-only lane (= 100%). Average free DNA remaining (Rel %) and standard deviation (SD) are presented below three independent repeats (i, ii, and iii).

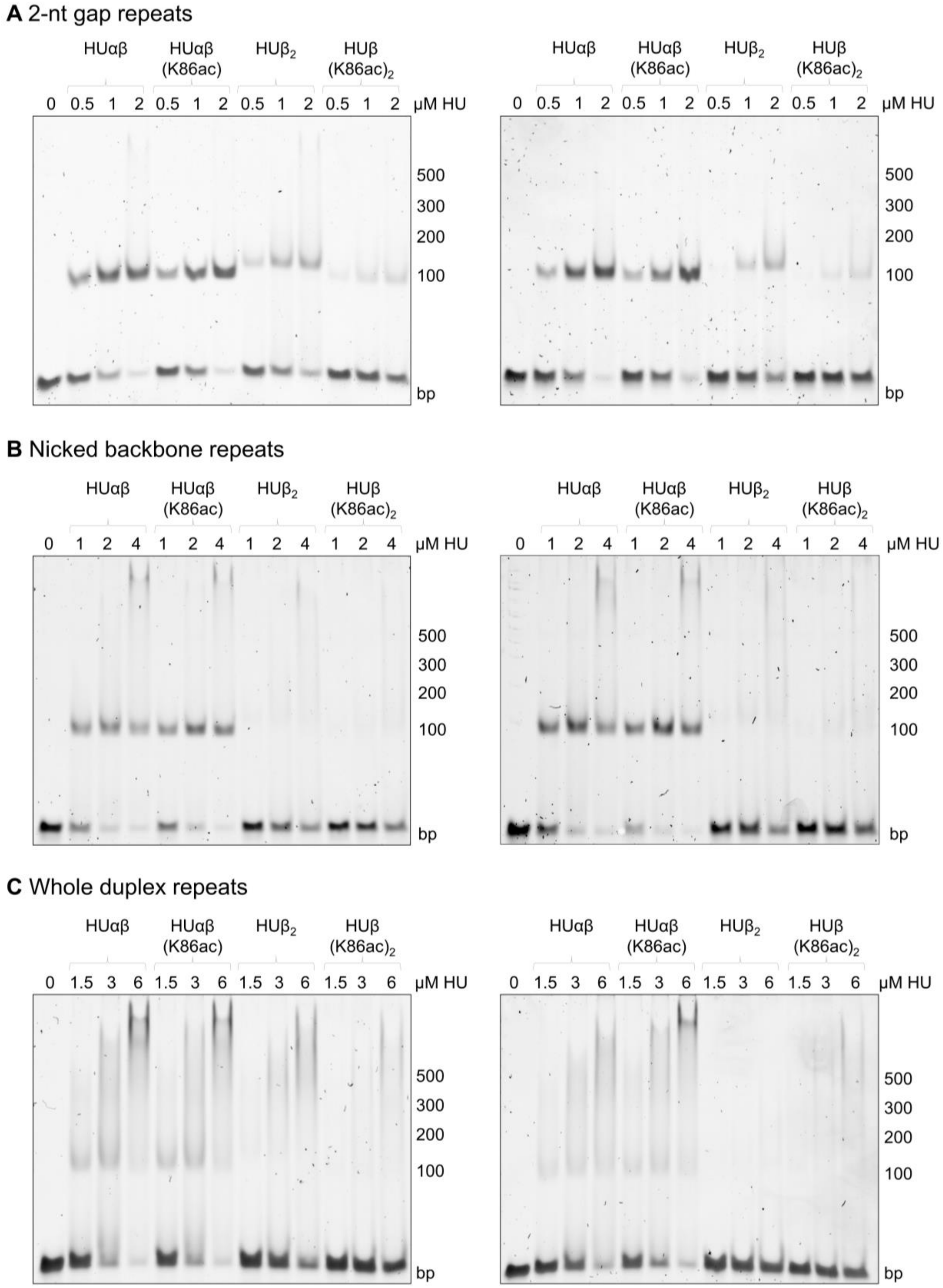

Supplementary Figure 3. Repeats of EMSAs from **Figure 4** of the main manuscript. (**A**) EMSA comparing the effect of acetylation on the HUβ_2 homodimer vs the HUαβ heterodimer when interacting with a 30 bp DNA fragment containing a 2-nucleotide gap. Repeats of the representative

figure in panel **A** of **Figure 4** of the main manuscript. (**B**) EMSA comparing the effect of acetylation on the HUβ_2 homodimer vs the HUαβ heterodimer when interacting with a 30 bp with a nicked phosphate backbone. Repeats of the representative figure in panel **B** of **Figure 4** of the main manuscript. (**C**) EMSA comparing the effect of acetylation on the HUβ_2 homodimer vs the HUαβ heterodimer when interacting with a 30 bp DNA fragment with a complete double-stranded duplex. Repeats of the representative figure in panel **C** of **Figure 4** of the main manuscript.

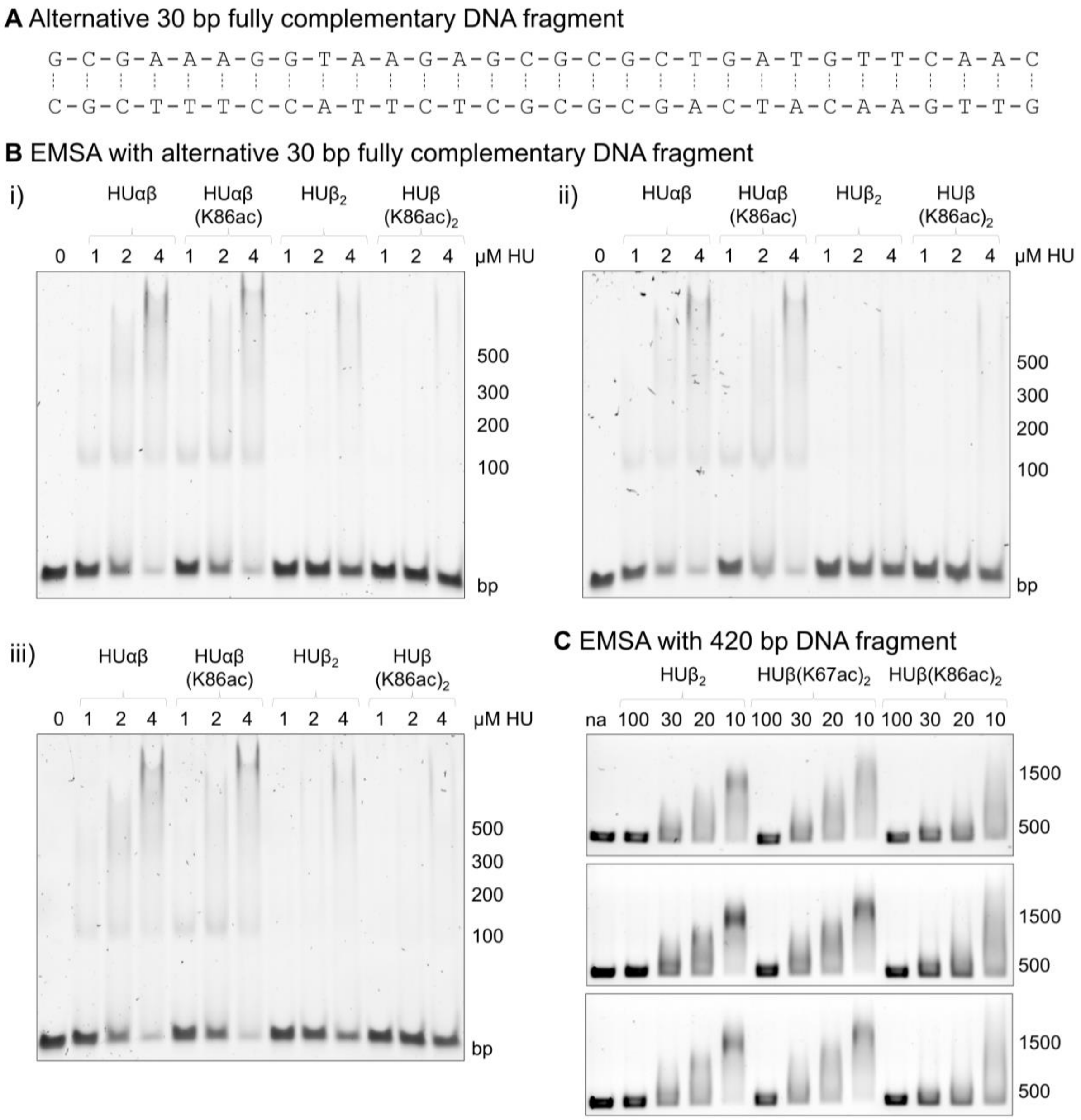

Supplementary Figure 4. EMSA with alternative 30 bp fully complementary DNA fragment and 420 bp DNA fragment. (**A**) Sequence of alternative fully complementary 30 bp DNA fragment. The phosphate backbone is indicated by a solid line and hydrogen bonds between base pairs are indicated by dashed lines. (**B**) EMSA comparing acetylation of Lys86 of HUβ_2 homodimer vs the HUαβ

heterodimer when interacting with an alternative 30 bp DNA fragment. Three repeats (i, ii and iii) are shown. (**C**) EMSA comparing acetylation of $HU\beta_2$ when interacting with a 420 bp DNA fragment.

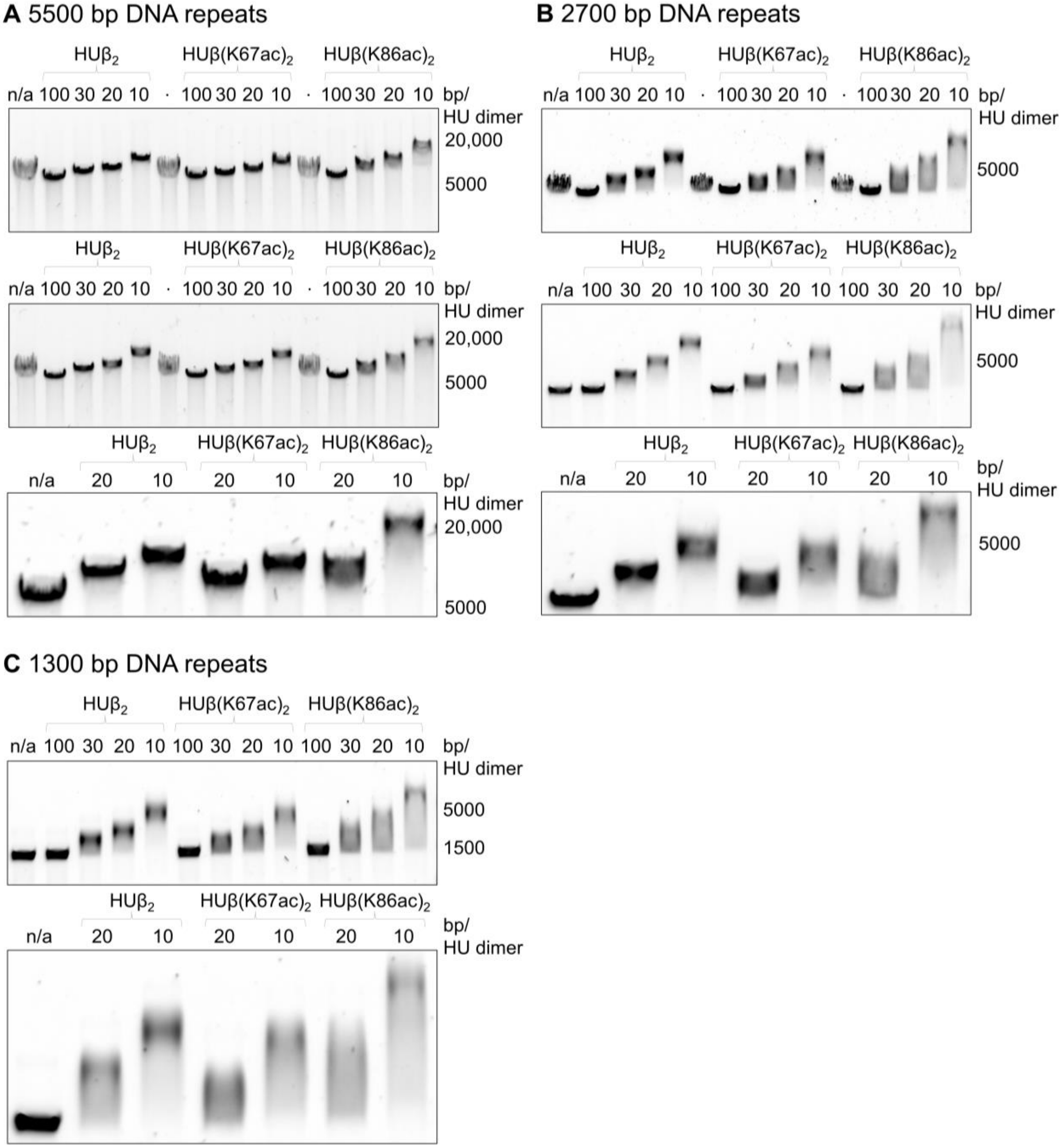

Supplementary Figure 5. Repeats of EMSAs from **Figure 4** of the main manuscript. **(A)** EMSA investigating the effect of acetylation of the $HU\beta_2$ homodimer on interaction with a 5500 bp linear DNA fragment. Repeats of the representative figure in **Figure 4D i)** of the main manuscript. (**B**) EMSA investigating the effect of acetylation of the $HU\beta_2$ homodimer on interaction with a 2700 bp linear DNA fragment. Repeats of the representative figure in **Figure 4D ii)** of the main manuscript.

(**C**) EMSA investigating the effect of acetylation of the HUβ_2 homodimer on interaction with a 1300 bp linear DNA fragment. Repeats of the representative figure in **Figure 4D iii)** of the main manuscript.

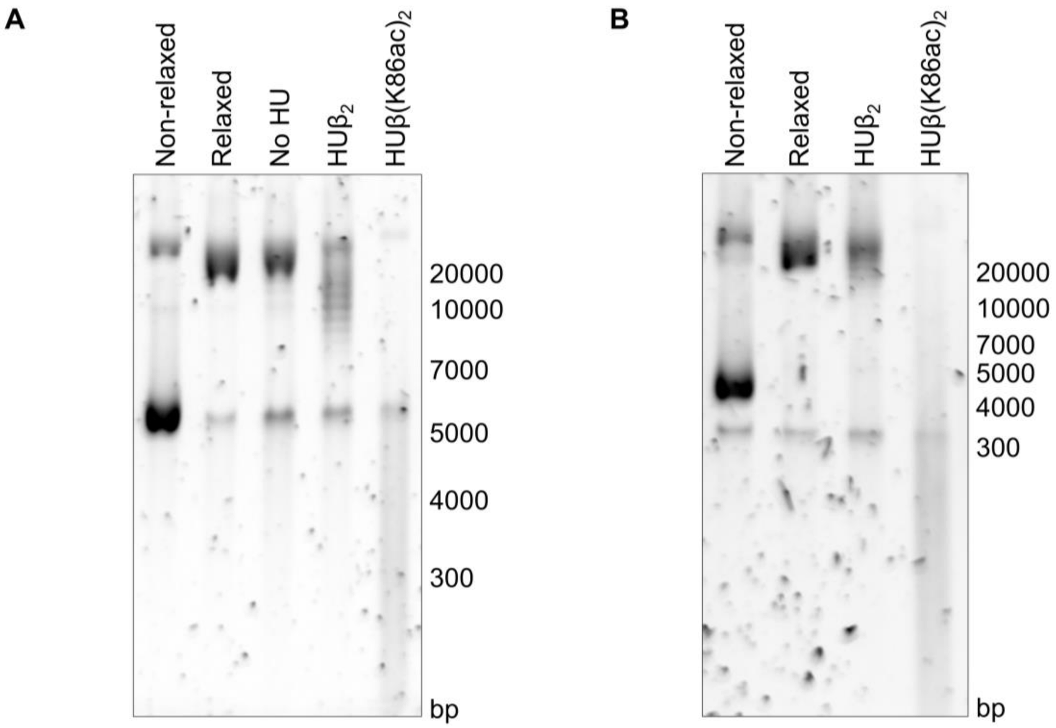

Supplementary Figure 6. Effect of acetylation at Lys86 on DNA supercoiling ability of HUβ_2 in the presence of topoisomerase I. Two repeats, **A** and **B**, are shown. A miniprepped 5500 bp plasmid (non-relaxed) was relaxed with *E. coli* topoisomerase I then purified (relaxed). The relaxed DNA was then incubated with HUβ_2, or HUβ(K86ac)$_2$ in the presence of calf thymus topoisomerase I. The samples were treated with proteinase K before electrophoresis.

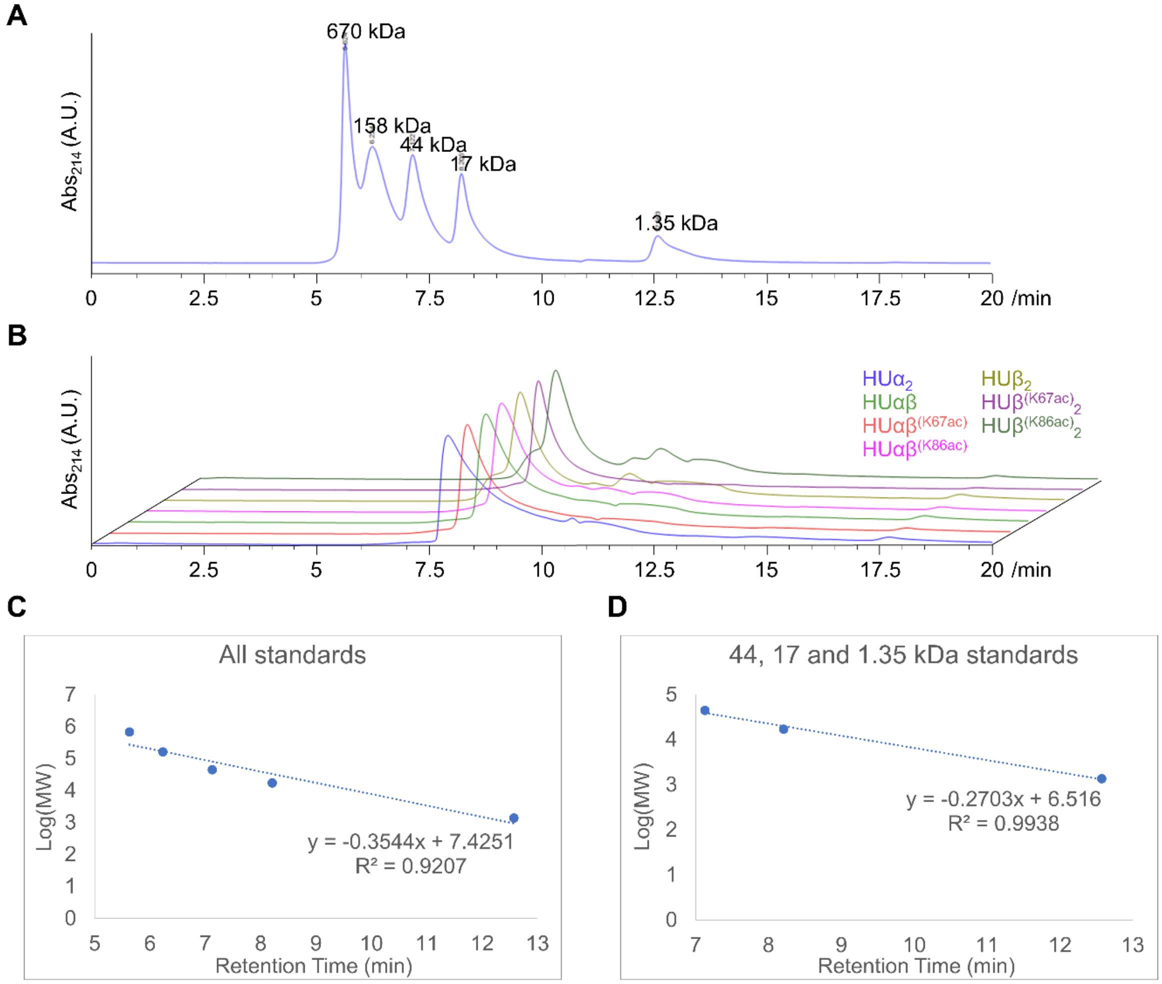

Supplementary Figure 7. Analytical size exclusion high-performance liquid chromatography. (**A**) Analytical size exclusion chromatography of Bio-Rad Gel Filtration standards. The absorbance at 214 nm is plotted against the retention time of each of the five protein standards. The known molecular weights of the standards are 670, 158, 44, 17 and 1.35 kDa. (**B**) Analytical size exclusion chromatography of HU proteins. The absorbance at 214 nm is plotted against the retention time. (**C**) A standard curve generated from the retention times of the 5 protein standards in A and the log of their molecular weights. (**D**) A standard curve generated from the retention times of the 44, 17 and 1.35 kDa standards and the log of their molecular weights. For estimated molecular weight calculations, please see **Supplementary Tables 7 - 9**.

2.2 Supplementary Tables

Supplementary Table 1. Calculations of free DNA remaining for EMSAs of HUβ$_2$ homodimers incubated with a 30 bp DNA fragment containing a 2-nucleotide gap. Average and standard deviation were calculated for three independent repeats. A single outlier was omitted from the calculations and is annotated in red.

	HUβ$_2$				HUβ(K67ac)$_2$				HUβ(K86ac)$_2$			
µM HU	0.5	1	2	4	0.5	1	2	4	0.5	1	2	4
Repeat 1	84	37	21	12	77	59	32	15	86	87	74	57
Repeat 2	93	82	29	11	83	52	28	14	97	93	69	41
Repeat 3	87	77	24	12	78	65	32	15	89	81	70	49
Average	88	80	25	12	79	59	31	15	91	87	71	49
Standard Deviation	4.6	3.5	4.0	0.6	3.2	6.5	2.3	0.6	5.7	6.0	2.7	8.0

Supplementary Table 2. Calculations of free DNA remaining for EMSAs of HUαβ heterodimers incubated with a 30 bp DNA fragment containing a 2-nucleotide gap. Average and standard deviation were calculated for three independent repeats.

	HUαβ				HUαβ(K67ac)				HUαβ(K86ac)			
µM HU	0.5	1	2	4	0.5	1	2	4	0.5	1	2	4
Repeat 1	69	27	5	4	68	35	5	4	70	45	10	8
Repeat 2	78	39	7	2	68	35	5	4	68	41	5	5
Repeat 3	82	61	9	2	77	48	23	9	79	58	12	2
Average	76	42	7	3	71	39	11	6	72	48	9	5
Standard Deviation	6.7	17.2	2.0	1.2	5.2	7.5	10.4	2.9	5.9	8.9	3.6	3.0

Supplementary Table 3. Calculations of free DNA remaining for EMSAs comparing the effects of acetylation on HUβ_2 homodimers vs HUαβ heterodimers incubated with a 30 bp DNA fragment containing a 2-nucleotide gap. Average and standard deviation were calculated for three independent repeats.

	HUαβ			HUαβ(K86ac)			HUβ_2			HUβ(K86ac)$_2$		
µM HU	0.5	1	2	0.5	1	2	0.5	1	2	0.5	1	2
Repeat 1	72	36	9	77	52	9	74	52	27	89	85	60
Repeat 2	64	26	4	71	42	3	66	48	20	75	76	67
Repeat 3	83	51	8	80	56	15	92	75	42	94	84	72
Average	73	38	7	76	50	9	77	58	30	86	82	66
Standard Deviation	9.5	12.6	2.7	4.6	7.2	6.0	13.3	14.6	11.2	9.6	4.9	6.0

Supplementary Table 4. Calculations of free DNA remaining for EMSAs comparing the effects of acetylation on HUβ_2 homodimers vs HUαβ heterodimers incubated with a 30 bp DNA fragment with a nicked phosphate backbone. Average and standard deviation were calculated for three independent repeats.

	HUαβ			HUαβ(K86ac)			HUβ_2			HUβ(K86ac)$_2$		
µM HU	1	2	4	1	2	4	1	2	4	1	2	4
Repeat 1	45	11	6	16	4	3	60	57	27	69	62	46
Repeat 2	52	13	9	54	9	5	98	65	35	102	91	71
Repeat 3	42	13	8	44	15	6	79	58	37	81	68	48
Average	46	12	8	38	9	5	79	60	36	84	74	47
Standard Deviation	5.1	1.2	1.5	19.7	5.5	1.5	19.0	4.4	1.4	16.7	15.3	1.4

Supplementary Table 5. Calculations of free DNA remaining for EMSAs comparing the effects of acetylation on HUβ$_2$ homodimers vs HUαβ heterodimers incubated with a 30 bp fully complementary DNA fragment. Average and standard deviation were calculated for three independent repeats.

	HUαβ			HUαβ(K86ac)			HUβ$_2$			HUβ(K86ac)$_2$		
μM HU	1.5	3	6	1.5	3	6	1.5	3	6	1.5	3	6
Repeat 1	84	37	14	76	36	11	90	68	51	82	86	70
Repeat 2	83	50	7	79	50	10	94	77	45	89	86	73
Repeat 3	89	76	21	81	42	14	100	89	80	78	73	67
Average	85	54	14	79	43	12	95	78	59	83	82	70
Standard Deviation	3.2	19.9	7.0	2.5	7.0	2.1	5.0	10.5	18.7	5.6	7.5	3.0

Supplementary Table 6. Calculations of free DNA remaining for EMSAs comparing the effects of acetylation on HUβ$_2$ homodimers vs HUαβ heterodimers incubated with an alternative 30 bp fully complementary DNA fragment. Average and standard deviation were calculated for three independent repeats.

	HUαβ			HUαβ(K86ac)			HUβ$_2$			HUβ(K86ac)$_2$		
μM HU	1	2	4	1	2	4	1	2	4	1	2	4
Repeat 1	88	59	17	85	61	15	85	75	48	82	88	75
Repeat 2	82	43	20	94	55	19	100	99	81	100	96	79
Repeat 3	92	64	15	93	60	22	100	98	74	100	98	91
Average	87	55	17	91	59	19	95	91	68	94	94	82
Standard Deviation	5	11	3	5	3	4	9	14	17	10	5	8

Supplementary Table 7. Retention times of Bio-Rad Gel Filtration Standards. The retention times of three repeats are provided alongside the known molecular weights of the proteins. The log of the molecular weights were plotted against the average retention times to generate a calibration curve (**Supplementary Figure 7A** and **B**).

	Retention Time (min)				MW (Da)	Log(MW)
	1	2	3	Average		
Thyroglobulin	5.636	5.626	5.624	5.629	670000	5.826
y-globulin	6.242	6.232	6.224	6.233	158000	5.199
Ovalbumin	7.135	7.125	7.122	7.127	44000	4.643
Myoglobin	8.215	8.209	8.205	8.210	17000	4.230
Vitamin B12	12.595	12.573	12.563	12.577	1350	3.130

Supplementary Table 8. Verifying standard curve molecular weight estimations. The line of best fits generated in the calibration curves (**Supplementary Figure 7A** and **B**) were used to calculate the molecular weights of the protein standards based on their retention times. This calculated value was compared to the known molecular weight of the standards. Equation 2 provides more accurate estimations of molecular weight at lower molecular weight ranges **(1350 – 44000 Da)** and was used for further analyses as the estimated molecular weights of HU protein dimers are within this range (*ca.* 24000 Da).

	MW (Da)	Log(MW)	Average RT (min)	Equation 1		Equation 2	
				Log(MW)	MW (Da)	Log(MW)	MW (Da)
Thyroglobulin	670000	5.826	5.629	5.430	269340	4.995	98758
y-globulin	158000	5.199	6.233	5.216	164529	4.831	67813
Ovalbumin	44000	4.643	7.127	4.899	79282	4.589	38858
Myoglobin	17000	4.230	8.210	4.516	32779	4.297	19812
Vitamin B12	1350	3.130	12.577	2.968	929	3.116	1307

Supplementary Table 9. Molecular weight estimations of HU proteins based on their retention times in analytical size exclusion chromatography. A standard curve based on the retention times of protein standards of known molecular weights (**Supplementary Figure 7D**, **Supplementary Tables 7-8**) was used to estimate the molecular weight of HU proteins. The estimations suggest that the HU proteins exist in their dimeric forms.

	RT (min)	Log (MW)	Calculated MW (Da)	Expected dimer MW	Expected monomer MW
HUα_2	7.908	4.378	23904	20716	10358
HU$\alpha\beta$	7.952	4.367	23258	20649	10358+10291
HU$\alpha\beta^{(K67ac)}$	7.94	4.370	23432	20691	10358+10333
HU$\alpha\beta^{(K86ac)}$	7.902	4.380	23993	20691	10358+10333
HUβ_2	7.915	4.377	23800	20582	10291
HU$\beta^{(K67ac)}{}_2$	7.916	4.376	23785	20666	10333
HU$\beta^{(K86ac)}{}_2$	7.901	4.380	24008	20666	10333
